# Acute effects of focused ultrasound-induced increases in blood-brain barrier permeability on rat microvascular transcriptome

**DOI:** 10.1038/srep45657

**Published:** 2017-04-04

**Authors:** Dallan McMahon, Reina Bendayan, Kullervo Hynynen

**Affiliations:** 1 University of Toronto, Department of Medical Biophysics, Toronto, M4N 3M5, Canada; 2 Sunnybrook Research Institute, Toronto, M4N 3M5, Canada; 3 University of Toronto, Department of Pharmaceutical Sciences, Toronto, M5S 3M2, Canada; 4 University of Toronto, Institute of Biomaterials and Biomedical Engineering, Toronto, M5S 3G9, Canada

## Abstract

Therapeutic treatment options for central nervous system diseases are greatly limited by the blood-brain barrier (BBB). Focused ultrasound (FUS), in conjunction with circulating microbubbles, can be used to induce a targeted and transient increase in BBB permeability, providing a unique approach for the delivery of drugs from the systemic circulation into the brain. While preclinical research has demonstrated the utility of FUS, there remains a large gap in our knowledge regarding the impact of sonication on BBB gene expression. This work is focused on investigating the transcriptional changes in dorsal hippocampal rat microvessels in the acute stages following sonication. Microarray analysis of microvessels was performed at 6 and 24 hrs post-FUS. Expression changes in individual genes and bioinformatic analysis suggests that FUS may induce a transient inflammatory response in microvessels. Increased transcription of proinflammatory cytokine genes appears to be short-lived, largely returning to baseline by 24 hrs. This observation may help to explain some previously observed bioeffects of FUS and may also be a driving force for the angiogenic processes and reduced drug efflux suggested by this work. While further studies are necessary, these results open up intriguing possibilities for novel FUS applications and suggest possible routes for pharmacologically modifying the technique.

Over the past 15 years, focused ultrasound (FUS) has emerged as a viable method for noninvasively inducing targeted and transient increases in BBB permeability. On of the main goals of this technique is to aid in drug delivery to the brain, a feat that continues to hinder the development of effective treatments for many neurological disorders^1^. This particular effect of FUS is achieved by intravenously administering a microbubble (MB) contrast agent at the onset of sonication. As intravascular MBs pass through the focus of the ultrasound beam, they oscillate, exerting a variety of forces on the vascular walls^2^. This mediates a temporary increase in transcellular transport^3^ and a widening of tight junctions^4^, allowing increased paracellular diffusion. Magnetic resonance imaging-guided FUS (MRIgFUS) has been shown to enhance the delivery of several therapeutic agents to the brain^5^,^6^,^7^,^8^, as well as induce other bioeffects, including increases in hippocampal neurogenesis^9^,^10^ and reduction in Alzheimer’s disease (AD)-like pathology^11^,^12^,^13^.

Central nervous system (CNS) vascular endothelium actively participates in signaling within the neurovascular unit, supporting neurogenesis, protecting neurons against stress, and mediating homeostasis^14^. When stimulated by physical stresses, the endothelium produces proinflammatory cytokines and chemokines which act to protect the brain from further damage, however, if this inflammatory response becomes chronic, neuroinflammation is magnified and secondary injury can occur^15^. The work described here is focused on the study of brain microvessels, as this is the site of increased permeability following FUS and experiences the largest magnitude of stress during sonication^2^. Given the relatively small proportion of total brain mass occupied by vasculature, limiting analysis to this tissue reduces the risk of significant changes being obscured by other tissues. Additionally, previous work has shown that the response to FUS is not homogeneous across cell types;^11^ thus, a more focused approach must be taken to glean useful information about the biological effects of sonication.

While the utility of FUS as a method of modulating BBB permeability has been thoroughly demonstrated in a variety of preclinical models^5^,^16^,^17^,^18^,^19^ and in human patients^20^, there remains a large gap in our knowledge regarding the biological events that follow sonication. Studies have thus far provided information relating to the routes of leakage from the circulation into the brain^3^,^4^,^21^ and have demonstrated examples of behavioural^12^,^22^,^23^,^24^, cellular^11^,^12^,^25^, and protein expression^4^,^26^,^27^ changes which may be related to the observed increases in BBB permeability. However, detailed information regarding the mechanisms driving increased permeability and the restoration of BBB function following FUS, as well as a full characterization of its safety profile, are lacking.

The work presented here is the first in depth *in vivo* exploration of how brain microvasculature responds at a transcriptional level in the acute stages following sonication. Using a systematic genome-wide transcriptome screening approach, FUS-induced changes to the rat brain vasculome were mapped at 6 and 24 hrs post-sonication. The knowledge gained from this work may guide strategies to pharmacologically modify the technique, may help to explain previous observations, and guide future research into novel applications of FUS. Ultimately, a detailed characterization of the brain microvascular response to FUS treatment will aid in clinical translation, providing a tool in the treatment of neurological diseases.

## Materials and Methods

### Animals

**Figure 1:**
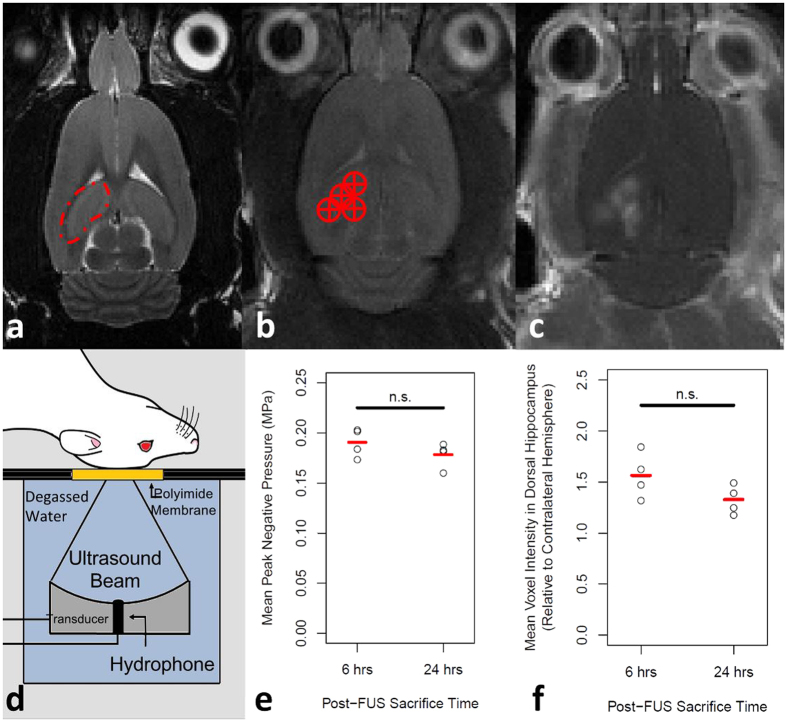
MRIgFUS. (**a**) The dorsal hippocampus (indicated by red dotted line) was (**b**) targeted from T2-weighted images (targets indicated by red circle with cross). (**d**) For sonication, the rat is positioned supine on an MRI compatible sled with the top of the skull coupled to a polyimide membrane. The bottom of the membrane was coupled to a tank filled with degassed, deionized water, housing the transducer/hydrophone assembly. The FUS procedure was applied as described in the methods section. (**e**) Mean peak negative pressure in the dorsal hippocampus following a software-triggered pressure drop were 0.19 MPa ± 0.02 MPa and 0.18 MPa ± 0.02 MPa (p = 0.246; unpaired, two-tailed, student’s t-test) for the 6 and 24 hrs post-FUS groups, respectively. Following FUS, (**c**) T1-weighted MR images were taken to confirm BBB permeability was increased. (**f**) When normalized to the contralateral hemisphere, the mean increase in dorsal hippocampal voxel intensity in the sonicated hemispheres was 56% ± 22% and 33% ± 14% in the 6 and 24 hrs post-FUS groups, respectively (p = 0.123; unpaired, two-tailed, student’s t-test).

Twelve male Sprague- Dawley rats, weighing 200–300 g, were used in this study (Taconic Biosciences, Germantown, NY, USA). Animals were housed in the Sunnybrook Research Institute animal facility (Toronto, ON, Canada) and had access to food and water *ad libitum*. To prepare animals for the FUS procedure, anesthesia was induced with isoflurane (5% at 1 L/min with oxygen), hair over the skull was removed with depilatory cream, and a 22-gauge catheter was placed in the tail vein. Anesthesia was maintained with a mixture of 80 mg/kg ketamine (Vétoquinol, Magny-Vernois, France) and 10 mg/kg xylazine (Bayer Inc., Toronto, ON, Canada) administered intramuscularly. Body temperature was maintained during FUS procedure with heated saline bags. Prior to sonication, animals were secured in a supine position on magnetic resonance imaging (MRI)-compatible sled. The top of the skull was coupled to a polyimide membrane with ultrasound gel (Fig. 1d). All animal procedures were approved by the Animal Care Committee at Sunnybrook Research Institute and are in accordance with the guidelines established by the Canadian Council on Animal Care.

### MRIgFUS Treatment

MRIgFUS was performed by one author (DM) using the RK100 system (FUS Instruments Inc., Toronto, ON, Canada). A spherically focused transducer (focal number = 0.8, external diameter = 75 mm, internal diameter = 20 mm), driven at a frequency of 551.5 kHz, was used for all sonications. The transducer was situated in a tank of degassed, deionized water and its movement was controlled with a motorized positioning system. The MRI-compatible sled was couple to the water tank during sonication (Fig. 1d). The spatial coordinates of the FUS positioning system were co-registered to that of a 7-Tesla MRI scanner (BioSpin 7030, Bruker, Billerica, MA, USA). This enables the targets of sonication to be chosen in the RK100 software from T2-weighted MR images (2000/60). Four locations were targeted per sonication (Fig. 1b). Eight locations were unilaterally targeted in one hemisphere of each animal.

Immediately prior to FUS, MBs (Definity, Lantheus Medical Imaging, North Billerica, MA, USA) were administered (20 μL/kg) via tail vein catheter (for simplicity, “FUS” will be used to describe sonication in the presence of MBs, unless explicitly stated). Ultrasound was delivered in 10 ms bursts with a pulse repetition frequency of 1 Hz for 120 seconds. The starting acoustic pressure was set at 0.128 MPa and increased by a 0.008 MPa increment every second. During sonication, acoustic emissions were monitored with an in-house manufactured polyvinylidene difluoride hydrophone located in a small perforation in the centre of the transducer (Fig. 1b). Once the ratio of signal above baseline at the first or second ultraharmonic frequency passed 3.5, the sonicating pressure was dropped by 50% and maintained at this level for the remainder of sonication. This algorithm is designed to calibrate pressure based on *in vivo* MB response, producing a more consistent and safe treatment^28^.

During sonication, a gadolinium-based contrast agent (Gadovist, Schering AG, Berlin, Germany) was injected into the venous circulation via tail vein catheter. T1-weighted images (500/10) were obtained immediately following FUS to confirm an increase in BBB permeability (Fig. 1c). Targets which did not demonstrate gadolinium contrast enhancement after the first sonication were treated a second time. All animals that received FUS displayed enhancement throughout either the left or right dorsal hippocampus, with the contralateral hemisphere remaining unaffected. Animals were sacrificed at 6 and 24 hrs post-sonication by transcardial perfusion with ice-cold saline, followed by 4% Evans blue dye (Sigma-Aldrich Corporation, St. Louis, MO, USA) in saline. Brains were snap frozen in liquid nitrogen and stored at −80 °C until processing.

### Tissue Processing

Brains were horizontally sectioned (10 μm) on a cryostat and mounted onto nuclease and nucleic acid free MembraneSlide NF 1.0 PEN slides (Zeiss, Göttingen, Germany). Eight sections throughout the dorsal hippocampus were collected from each brain. Mounted sections were stored up to 3 days at −80 °C before laser capture microdissection (LCM). Immediately prior to LCM, sections were briefly dehydrated in ethanol (ice-cold 95% for 30 s, ice-cold 100% for 30 s, and room temperature 100% for 30 s) and cleared in xylenes (twice at room temperature for 30 s). Sections were allowed to dry for 5 min in a fume hood prior to LCM.

### Microvessel Collection

**Figure 2:**
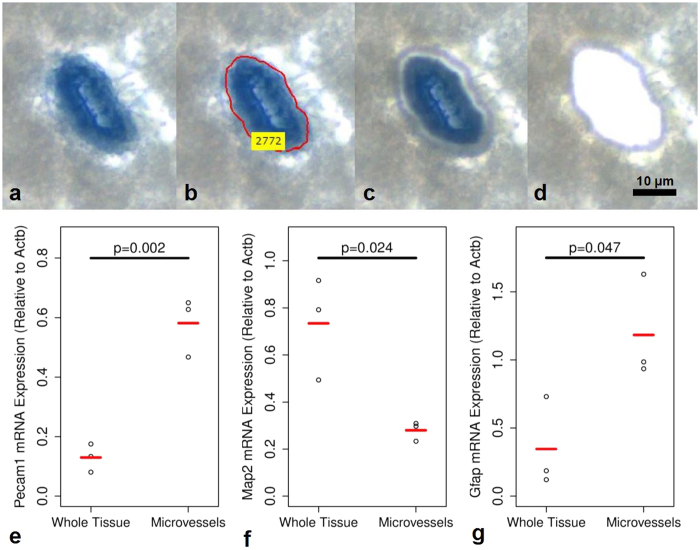
Laser capture microdissection and characterization of Evans blue perfused microvessels. Animals were transcardially perfused with ice-cold saline, followed by Evans blue dye, and the brain tissues were snap frozen in liquid nitrogen. The dye perfused blood vessels (**a**) were identified in an unbiased manner with an image analysis algorithm (**b**). These regions of interest were cut with a focused laser (**c**) and catapulted into a collection vessel (**d**) for subsequent RNA extraction. Relative to LCM collected whole tissue, LCM collected microvessel samples contained 348% (p = 0.002) and 242% (p = 0.042) higher levels of Pecam1 (**e**) and Gfap (**g**), respectively, and 62% (p = 0.024) lower levels of Map2 (**f**). Unpaired, two-tailed, student’s t-tests were used to evaluate statistical significance.

Dorsal hippocampal microvascular samples were collected using a PALM Microbeam system (Zeiss, Göttingen, Germany). The non-contact nature of this technology minimizes risks of contamination. An image processing algorithm was developed (AxioVision 4.8.3 software, Zeiss, Göttingen, Germany) to select Evans blue dye perfused microvessels (<50 μm in diameter) to be collected in an unbiased manner based on RGB and size thresholds (Fig. 2a–d). Imaging and collection of microvessels were performed using a 40x objective. Approximately 1 000 000 μm^2^ of tissue was collected into AdhesiveCap 500 microcentrifuge tubes (Zeiss, Göttingen, Germany) per sample, comprising approximately 3000 microvessel segments and yielding 3–5 ng of total RNA. Collection times were limited to 4 hrs to minimize the degree of RNA degradation. RNA isolation was achieved using the PicoPure kit (Life Technologies Inc., Waltham, MA, USA) according to manufacturer’s instructions. Samples were treated with DNase (Qiagen, Hilden, Germany). RNA concentration and quality was assessed using the 2100 Bioanalyzer system with RNA 6000 Pico Kit (Agilent, Santa Clara, CA, USA). All samples had an RNA integrity number of 6.8 or higher (7.3 ± 0.3).

### Characterization of Microvessel Sample Composition

In a separate cohort of male Sprague Dawley rats not receiving FUS (n = 3), the composition of LCM collected microvessels was assessed by semi-quantitative polymerase chain reaction (PCR). The tissue processing and LCM collection protocols for these samples were identical to that described above. Samples containing microvessels were collected from 3 separate brains (~1 000 000 μm^2^ of tissue/sample); from the same tissue sections and region of the brain, an equal amount of whole tissue was collected by LCM. RNA was isolated, treated with DNase, and assessed as described above. Semi-quantitative PCR was used to assess the level of Pecam1 (endothelial cell marker), Map2 (mature neuronal marker), Gfap (astrocyte marker), and beta-actin (Actb) (housekeeping gene). The forward and reverse primers used are as follows: Pecam1, forward, 5′-CCGTGATAGTGAACAGCAAGGA-3′, and reverse, 5′-AGGATGCTACTGGCCTTGGAGA-3′; Map2, forward, 5′-CATACCACCAGCGGTTTGAGT-3′, and reverse, 5′-GCTGAGGAACTAAGGCAGCA-3′; Gfap, forward, 5′-CGCGGCACGAACGAGTCC-3′, and reverse, 5′-GTGTCCAGGCTGGTTTCTCG-3′; Actb, forward, 5′-AGGGAAATCGTGCGTGACAT-3′, and reverse, 5′-GCAGCTCAGTAACAGTCCGC-3′. SuperScript III one-step RT-PCR system with platinum taq (Life Technologies Inc., Waltham, MA, USA) was used with an annealing temperature of 58 °C for all PCR reactions except with Gfap primers (56 °C). A total of 35, 32, 35, and 30 PCR cycles were completed for Pecam1, Map2, Gfap, and Actb, respectively. PCR products were separated by electrophoresis on 2.0% agarose gels with TAE and ethidium bromide. All samples were run in triplicate. Gels were photographed under ultraviolet light using the MiniBIS Pro gel image analysis instrument (DNR Bio-Imaging Systems Ltd., Jerusalem, Israel). To assess the expression of Pecam1, Map2, and Gfap, the integrated density values of PCR product bands were normalized against Actb density. Unpaired, two-tailed, student’s t-tests were used to assess statistical significance.

### Microarray Processing and Analysis

Relative gene expression was assessed using the Affymetrix Rat 2.0 ST array (Santa Clara, CA, USA). Sample preparation was performed using GeneChip WT Pico Kit (Affymetrix, Inc., Santa Clara, CA, USA) with 500 pg of starting total RNA. Sample preparation and microarray processing was performed at The Centre for Applied Genomics (Toronto, ON, Canada). A total of 20 microvascular samples were analysed from 5 groups (n = 4/group); groups included, 6 hrs post-FUS ipsilateral hippocampus, 6 hrs post-FUS contralateral hippocampus, 24 hrs post-FUS ipsilateral hippocampus, 24 hrs post-FUS contralateral hippocampus, and rats receiving no FUS treatment.

All microarray data analysis was performed using the statistics program, R. Robust multi-array averaging was used for pre-processing (“oligo” package, Bioconductor), empirical bayes analysis (“limma” package, Bioconductor) to assess differential expression, and the Benjamini–Hochberg method to adjust for multiple comparisons. Microarray quality control included performing outlier detection on MA plots by computing Hoeffding’s statistic D on the joint distribution of A and M for each microarray (D < 0.02 for all microarrays). A gene was considered differentially expressed between two groups if the log2 fold-change was greater or less than 1.0 or −1.0, respectively, and had an adjusted p-value of less than 0.05. A total of 19105 genes were assessed for differential expression.

### Gene Ontology Over-representation and Geneset Enrichment Analysis

*ToPASeq* (Bioconductor) was used to identify gene ontology (GO) terms that were altered in microvessels by FUS treatment. For both over-representation analysis (ORA) and geneset enrichment analysis (GSEA), the GO sub-ontologies, *Biological Process* and *Molecular Function,* were assessed and the Benjamini–Hochberg method was used to adjust for multiple comparisons. ORA can be used to assess whether a subset of genes with related functions, GO terms, are enriched in a list of differentially expressed genes using a hypergeometric test. For this analysis, genes displaying significant changes between groups in relative expression were divided into up- and downregulated genes. An adjusted p-value of 0.001 was used as a threshold for significance. Similarly, GSEA can be used to analyse whether a significant proportion of genes that are part of a GO term fall in the extremes of a ranked list of genes. For this analysis, all of the genes assessed by microarray were ranked by log2 fold change of differential expression between groups and a normalized enrichment score (NES) was calculated for each GO term. The advantage of this technique is that it utilizes information from the entire microarray dataset to determine which GO terms are enriched and to what degree. An adjusted p-value of 0.05 and NES of less than −1.5 or greater than 1.5 were used as thresholds for significance.

### Quantitative Real Time Polymerase Chain Reaction

To confirm the differential gene expression observed in the microarray data, relative expression of selected genes were also assessed by quantitative real time-PCR (qRT-PCR). The gene-specific primers used are listed in Table 1. Amplified cDNA from the GeneChip WT pico kit was used as template. qRT-PCR was performed in triplicate on a CFX-96 real-time PCR detection system (BioRad Laboratories, Inc., Hercules, CA, USA), using SYBR green master mix (Thermo Fisher Scientific, Waltham, MA, USA). Relative gene expression of each transcript was determined by normalizing against Gapdh, using the ΔΔCt method. Following qRT-PCR, specificity of each gene amplicon was confirmed by melting curve analysis and gel electrophoresis.

## Results

### Characterization of LCM collected microvessels

The composition of LCM collected microvessels was assessed by semiquantitative PCR in a separate cohort of rats not receiving FUS. The expression of Pecam1, Map2, and Gfap was compared between LCM collected microvessels and LCM collected whole tissue as a measure of endothelial cell (EC), mature neuron, and astrocyte content, respectively (Fig. 2e–g). Relative to whole tissue, microvessel samples contained 348% (p = 0.002) and 242% (p = 0.042) higher levels of Pecam1 and Gfap, respectively, and 62% (p = 0.024) lower levels of Map2. Given the structural organization of the BBB, with vasculature being tightly ensheathed in astrocytic endfeet and innervated by excitatory neurons, an enrichment of Gfap and reduction (but not absence) of Map2 in LCM collected microvessel samples compared to whole brain tissue is logical. Increases in Gfap expression in isolated microvessels has been previously reported with other techniques^29^,^30^. Thus, LCM collected microvessel samples used in this work are highly enriched with brain microvasculature.

### Increased gadolinium contrast enhancement in dorsal hippocampus following FUS

BBB permeability was assessed following FUS by gadolinium contrast enhancement on T1-weighted MRI. Normalized to the contralateral hemisphere, the mean increase in dorsal hippocampal voxel intensity in the sonicated hemispheres (Fig. 1f) was 56% ± 22% and 33% ± 14% in the 6 and 24 hrs post-FUS groups, respectively (p = 0.123). This similarity between groups in gadolinium contrast enhancement was mirrored by the mean acoustic energy delivered to the dorsal hippocampus of each group. For the 6 and 24 hrs post-FUS groups, respectively, the mean peak negative pressure in the dorsal hippocampus following a software-triggered pressure drop (Fig. 1e) were 0.19 MPa ± 0.02 MPa and 0.18 MPa ± 0.02 MPa (p = 0.246). These data suggest that the FUS treatments were similar between time points and resulted in a similar increase in BBB permeability.

### Microarray gene expression

**Figure 3:**
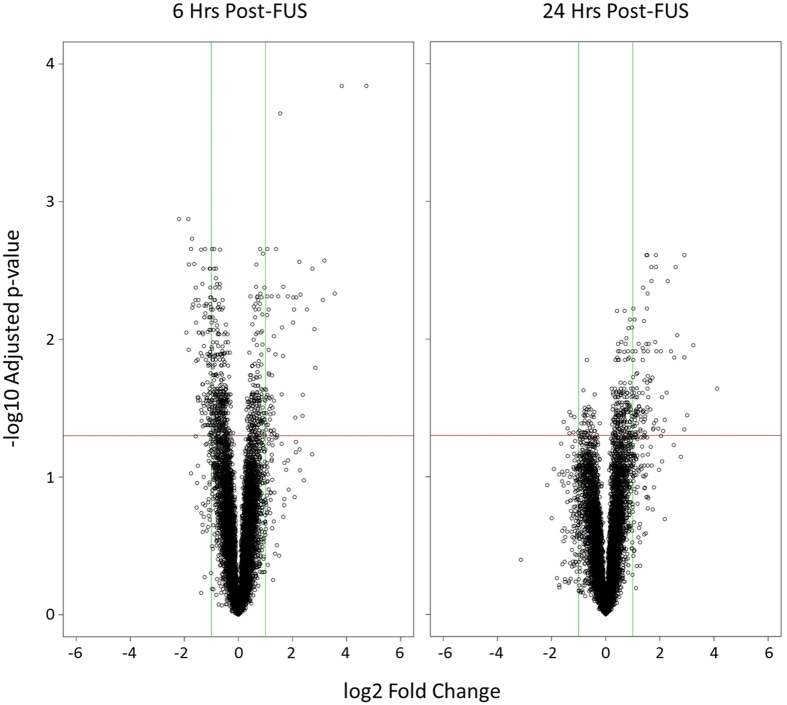
Volcano plots of differential gene expression. Relative gene expression in hippocampal microvessels was compared between the sonicated and non-sonicated hemispheres at 6 and 24 hrs post-FUS. A positive log2 fold change indicates increased relative expression in sonicated microvessels compared to contralateral hemisphere. Green vertical lines indicate a log2 fold change of 1.0 or −1.0. Red horizontal lines indicate a -log10 adjusted p-value of 1.30, corresponding to an adjusted p-value of 0.05.

Differential gene expression in dorsal hippocampal microvessels was assessed between samples collected in the sonicated and contralateral hemispheres at 6 and 24 hrs following FUS (Fig. 3). 19105 genes were included in analysis; 60 genes were upregulated in the sonicated hemisphere after 6 hrs (Supplementary Table 1), while 109 were downregulated (Supplementary Table 2). At the 24 hr time point, 101 and 8 genes were up and downregulated, respectively (Supplementary Tables 1 and 2). When comparing samples collected from the hemisphere contralateral to sonication at 6 or 24 hrs post-FUS and samples from animals not receiving the FUS procedure (sham animals), no transcripts displayed significant changes in expression at either time point investigated, suggesting that non-local effects of FUS on transcription are minimal (Supplementary Figure 1).

### Transient upregulation of inflammatory-related genes following FUS

**Figure 4:**
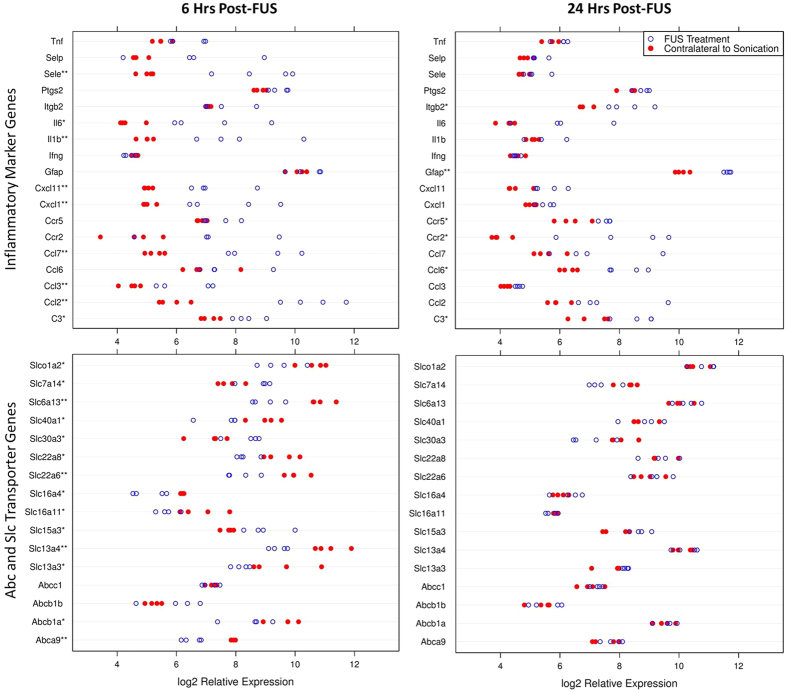
Dotplots of inflammatory marker and Abc and Slc gene expression. Relative gene expression in hippocampal microvessels was compared between the sonicated (open circle) and non-sonicated (close red circle) hemispheres at 6 and 24 hrs post-FUS. Relative expression of selected genes are displayed for each microvascular sample. *Indicates p < 0.05. **Indicates p < 0.01.

A consistent upregulation in many genes related to an acute inflammatory response was detected in dorsal hippocampal microvessels at 6 hrs post-FUS. Genes of note include, Sele, Cxcl1, Ccl3, and Ccl2, displaying log2 fold changes of 3.82 (p < 0.001), 2.74 (p = 0.003), 1.83 (p = 0.005), and 4.73 (p < 0.001), respectively, compared to the contralateral hemisphere (Fig. 4). There was a return to baseline in the expression of many of these genes by 24 hrs following sonication. For the same genes listed above, there is a non-significant log2 fold change of 0.40 (p = 0.650), 0.47 (p = 0.621), 0.44 (p = 0.495), and 1.70 (p = 0.082), respectively, at 24 hrs post-FUS (Table 2). It appears that FUS induces an acute inflammatory response that largely returns to baseline or is greatly dampened at 24 hrs post-sonication, however, there were inflammatory markers which had their gene expression remain significantly upregulated at 24 hrs following sonication including, C3, Ccl6, Gfap, and Itgb2. These genes displayed log2 fold changes of 1.55 (p = 0.023), 1.94 (p = 0.045), 1.54 (p = 0.003), and 1.38 (p = 0.034), respectively (Fig. 4). While these genes have involvement in inflammation, some may be indicative of immune cell infiltration^31^,^32^.

The observed changes in individual transcripts related to a transient inflammatory response are reiterated by gene set enrichment analysis (GSEA). GO terms related to inflammation, such as “chronic inflammatory response” (GO:0002544) and “inflammatory response to antigenic stimulus” (GO:0002437), show significant enrichment at 6 hrs post-FUS with NESs of 2.27 (p = 0.015) and 2.18 (p = 0.015), respectively. At 24 hrs following sonication, the NESs for these same GO terms are 1.99 (p = 0.009) and 2.13 (p = 0.009), respectively, suggesting a plateau or reduction in these inflammatory responses at the later time point. GSEA and ORA of genes displaying increased expression in dorsal hippocampal microvessels following FUS show considerable overlap in GO terms related to a transient inflammatory response (Supplementary Table 6). There is, however, a consistent reduction in ORA significance levels at the later time point for these GO terms.

### FUS induces downregulation of BBB transporter genes

Downregulation in the expression of BBB transporter genes was observed at 6 hrs following sonication for several members of the Abc and Slc families of transporters including, Abcb1a, Abca9, Slc22a6, Slc22a8, and Slc6a13 with log2 fold changes of −1.14 (p = 0.042), −1.38 (p = 0.002), −1.83 (p = 0.003), −1.19 (p = 0.028), and −1.85 (p = 0.001), respectively (Fig. 4). Expression of these transcripts returned to baseline by 24 hrs, displaying non-significant log2 fold changes of 0.08 (p = 0.927), 0.26 (p = 0.548), 0.18 (p = 0.794), −0.01 (p = 0.997), and 0.24 (p = 0.671), respectively (Table 2).

**Figure 5:**
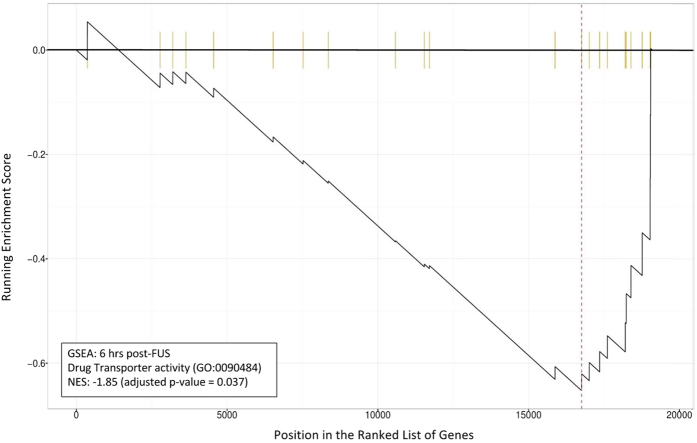
GSEA for GO term “drug transporter activity” 6 hrs post-FUS. The running enrichment score for “drug transporter activity” (GO:0090484) is depicted in relation to a list of genes ranked by log2 fold change at 6 hrs post-FUS. Each vertical yellow line indicates the location of a gene in the ranked list that is associated with this GO term. NES = −1.85 (adjusted p = 0.037). GO term are comprised of a list of genes whose function are related. Thus, the non-random distribution of these genes within the ranked list is indicative of drug transporter suppression.

These changes in the expression of genes related to transport across the BBB are reiterated in GSEA. NES at 6 hrs following FUS for the GO term “drug transporter activity” (GO:0090484) was −1.85 (p = 0.037), indicating significant suppression (Fig. 5). At 24 hrs post-FUS, NES partially normalizes to 0.93 (p = 0.754). In addition to drug transporter activity, various other GO terms related to BBB transporter activity display significantly negative NESs at 6 hrs post-FUS. These include, “amino acid transmembrane transporter activity” (GO:0015171), “monocarboxylic acid transmembrane transporter activity” (GO:0008028), “active transmembrane transporter activity” (GO:0022804), and “secondary active transmembrane transporter activity” (GO:0015291), with NESs of −1.59 (p = 0.037), −1.97 (p = 0.025), −1.63 (p = 0.025), and −1.65 (p = 0.025), respectively. NESs for these same GO terms at 24 hrs post-FUS are −1.48 (p = 0.080), 1.06 (p = 0.592), −1.33 (p = 0.041), and −1.34 (p = 0.065), respectively, indicating a return to baseline (Table 3).

An examination of individual transcript expression and GSEA would suggest that several transporters in the BBB may have reduced activity at 6 hrs following sonication, with a return to baseline at 24 hrs; however, GSEA indicates that BBB ion transporter activity at 24 hrs post-FUS may become suppressed. GO terms related to ion transporter activity displaying suppression include, “potassium ion transmembrane transporter activity” (GO:0015079), “calcium ion transmembrane transporter activity” (GO:0015085), “sodium ion transmembrane transporter activity” (GO:0015081), “chloride transmembrane transporter activity” (GO:0015108), and “ion transmembrane transporter activity” (GO:0015075), with NESs of −2.44 (p = 0.020), −2.37 (p = 0.020), −1.93 (p = 0.020), −1.93 (p = 0.020), and −2.14 (p = 0.022), respectively (Table 3).

### Upregulation of angiogenesis-related genes following FUS

At 6 hrs post-FUS, there was a significant increase in the expression of several genes related to angiogenesis, including Serpine1, Ccl2, Egr3, and Mmp9, with log2 fold changes of 2.37 (p = 0.036), 4.73 (p < 0.001), 1.23 (p = 0.005), and 2.53 (p = 0.006), respectively. At the later time point, Itgb2, Ccr5, Lcn2, Lgals3, and Ccr2 display significant increases in expression, with log2 fold changes of 1.38 (p = 0.034), 1.15 (p = 0.036), 2.91 (p = 0.003), 2.29 (p = 0.004), and 4.12 (p = 0.023), respectively (Table 2).

GSEA of differential gene expression between sonicated and contralateral hemisphere dorsal hippocampal microvessels indicates that angiogenesis related pathways may be activated by FUS. GO terms, such as “positive regulation of angiogenesis” (GO:0045766; Fig. 6), “positive regulation of vasculature development” (GO:1904018), “blood vessel morphogenesis” (GO:0048514), and “positive regulation of blood vessel endothelial cell migration” (GO:0043536), display NESs of 2.48 (p = 0.009), 2.48 (p = 0.009), 2.34 (p = 0.009), and 1.95 (p = 0.009), respectively at 24 hrs port-FUS. At 6 hrs following FUS, NES for “vascular endothelial growth factor production” (GO:0010573) is 2.29 (p = 0.015) (Table 3).

ORA also gives some indication that angiogenic processes are upregulated following FUS. At 6 hrs post-sonication, there is a significant over-representation of differentially expressed genes that are part of the following GO terms, “regulation of angiogenesis” (GO:0045765; p < 0.001), “positive regulation of vasculature development” (GO:1904018; p < 0.001), “regulation of vascular endothelial growth factor production” (GO:0010574; p < 0.001), “blood vessel morphogenesis” (GO:0048514; p < 0.001), and “vascular endothelial growth factor production” (GO:0010573; p < 0.001). At 24 hrs following sonication, there is a significant over-representation of differentially expressed genes that are part of the “positive regulation of vasculature development” (GO:1904018; p < 0.001) and “positive regulation of angiogenesis” (GO:0045766; p < 0.001) GO terms (Supplementary Table 5).

### Differential expression detected by microarray analysis strongly correlated to qRT-PCR results

Differential expression of selected transcripts, shown to be up or downregulated at one or both of the post-FUS time points by microarray analysis were assessed by qRT-PCR (Supplementary Table 6). Genes of interest included, Abcb1a, Ccl2, Cd74, Gfap, Itgb2, Lcn2, Serpine1, and Slc22a6. A strong correlation (r^2^ = 0.959) of log2 fold changes between analysis methods was observed (Supplementary Figure 2), adding confidence to the conclusions drawn from the microarray data. Regression analysis of differential expression detected by microarray analysis and qRT-PCR suggest that the microarray data may be a conservative estimate of differential gene expression.

## Discussion

An assessment of individual gene expression changes and bioinformatic analysis of microarray data presented here suggests that there is an acute inflammatory response in hippocampal microvessels following sonication. The stresses exerted on the vascular endothelium by oscillating MBs may trigger an increase in the transcription of proinflammatory cytokine genes. At 6 hrs post-sonication there were significant differences detected in the transcription of Ccl2, Ccl3, Ccl7, Cxcl1, Cxcl11, Il1b, and Il6 compared to microvessels in the contralateral hemisphere. Additionally, gene expression of several other indicators of inflammation were elevated at this early time point. If the observed changes in gene expression are in fact indicative of increased activation of vascular ECs and release of proinflammatory cytokines and chemokines, this would likely act to promote infiltration of leukocytes across the BBB, as evidenced by GSEA. Importantly, this inflammatory response seems to be greatly dampened by 24 hrs post-FUS, with a return to baseline in the gene expression of Ccl2, Ccl3, Ccl7, Cxcl1, Cxcl11, Pge2, Il1b, and Il6. Additionally, increased expression of Sele, indicating EC activation, returns to baseline by 24 hrs; expression of Vcam1 and Icam1 are not elevated at either time point.

In line with previous literature showing an increase in immunodetection at 4 and 15 days following sonication^11^, we report an increase in Gfap gene expression detected at 24 hrs post-FUS, indicative of astrocyte activation. While the chronic nature of reactive gliosis in several CNS pathologies can lead to inhibitory effects on neuroplasticity and regeneration, acute astrocyte activation can play a critical role in neuroprotection and regulation of homeostasis in acute ischemia and various types of stresses^33^. The astrocyte activation in endfeet surrounding microvessels suggested by this work may play an important role in restoring the extracellular environment in the CNS following FUS. Activation of astrocytes following FUS may be related to increased gene expression of cytokines by endothelial cells, as well as from astrocytes and other cells of the parenchyma^34^.

While chronic inflammation in the brain has detrimental effects, inducing necrosis, apoptosis, and pyroptosis^34^, acute inflammation can induce a wide spectrum of changes, some of which may be beneficial. The release of Ccl2 and Ccl3, as well as signalling through Ccr2 and Ccr5 can promote the migration, proliferation, differentiation, and survival of neural progenitor cells^35^. A transient and controlled level of neuroinflammation can also promote myelin debris clearance, myelin repair^36^, angiogenesis^37^, and amyloid beta (Aβ) plaque clearance^38^. When inflammation becomes chronic, however, the production of Tnfα, Il6, and reactive oxygen species within the CNS act to suppress neurogenesis and leads to apoptosis and neurodegenerative processes^35^.

An increase in the gene expression of Alox5ap 24 hrs following FUS may contribute to the resolution of inflammation by affecting the activity of 5-lox and subsequently the production of lipoxins and resolvins^39^. Additionally, increased expression of Timp1 has been found to attenuate inflammation in endothelium;^40^ Lyz2 has been implicated in reduction of Il1β, Il6, Tnfα, and Ccl2 production in human ECs;^41^ Slamf7 has been shown to inhibit the production of proinflammatory cytokines^42^. The increased gene expression of Timp1, Lyz2, Slamf7, and Alox5ap 24 hrs following FUS (Table 2) may contribute to the observed reduction in cytokine/chemokine gene expression, may reduce EC activation, and may mediate a resolution of inflammation.

Evidence of acute inflammation observed following FUS may be the driving force for many of the bioeffects previously reported, as well as some suggested from the results of this work. Hippocampal neurogenesis has been observed following FUS^9^,^10^, an effect that requires the presence of MBs and for the peak negative pressure of sonication to be sufficient to induce increases in the permeability of the BBB^10^. While neurogenesis can be induced by a wide variety of stimuli, the production of specific factors related to acute inflammation, such as Ccl2, Ccl3, and Tnfα, has been demonstrated to promote migration, proliferation, differentiation and survival of neural progenitor cells^35^. There is, however, a point at which inflammation has deleterious effects on neurogenesis. Chronic production and release of proinflammatory cytokines, like Il6, Il1β, Tnfα, and Ifng, suppress proliferation and survival of neural progenitor cells^35^.

The pattern of gene expression changes in microvessels reported here, with increased expression of Ccl2, Ccl3, Tnfaip2, and Tnfaip3 6 hrs following sonication and a return to baseline at 24 hrs, as well as no significant differences detected in the expression of Il6, Tnf, and Ifng at the latter time point, may create an environment that is favourable to neurogenesis. Although this hypothesis is yet to be tested, regardless of the driving mechanisms involved in FUS-induced neurogenesis, the fact that increased proliferation and survival is seen 18 days following sonication^9^ would suggest that chronic inflammation is not associated with FUS treatment. Consistent with this is the observation that nuclear localization of the transcription factor NF-kB, a marker of excessive, chronic inflammation, is not elevated as a result of ultrasound-mediated increases in BBB permeability^13^.

Based on the observed reduction in Abc and Slc transporter gene expression 6 hrs following sonication, and negative enrichment scores for several BBB transport-related GO terms, we hypothesize that FUS may induce a reduction in efflux across the BBB. Previous work by Cho *et al*. has shown that FUS induces a downregulation in immunodetection of P-gp 24 hrs post-sonication^43^. Results presented here are consistent with this finding, as there was a significant reduction in Abcb1a gene expression 6 hrs following FUS. Due to the well characterised role of P-gp in drug efflux from the brain and its contributions to drug resistance^44^, a reduction in its expression could act to increase the efficiency of FUS-mediated drug delivery by limiting efflux. Additionally, decreased gene expression of several members of the Slc family, including Slc22a6 and Slc22a8, were detected at 6 hrs following FUS. These genes code for Oat1 and Oat3, which have been implicated in the transport of a variety of therapeutic agents, including antiretroviral drugs^45^ and chemotherapeutics^46^,^47^,^48^, amongst others^49^,^50^. While Oat1 and 3 have primarily been characterized as “uptake transporters”, several reports describe their roles in drug efflux across the BBB (brain to blood transport)^51^,^52^,^53^. If FUS induces a transient downregulation in the expression of drug efflux transporters in the BBB, this could act to increase the effectiveness of FUS-mediated therapeutic agent delivery. It would also be an important consideration when developing dosing strategies that avoid toxicity following sonication.

Inflammation has previously been reported to induce a rapid reduction in Abcb1a gene expression in the rat brain, peaking at 6 hrs following the delivery of an inflammatory stimulus^53^,^54^. At the protein level, HIV-1 associated brain inflammation has been shown to induce a downregulation in P-gp expression both *in vitro*, in primary cultures of rat astrocytes^55^, and *in vivo*, in rats^56^. Pro-inflammatory cytokines have also been shown to reduce protein expression and functionality of P-gp in the BB^57^. Likewise, lipopolysaccharide (LPS)-induced inflammation has been shown to reduce expression of Slc22a6 and Slc22a8 in rat liver starting at 6 hrs following administration^58^. Given the concurrent increase and decrease in pro-inflammatory cytokine and transporter gene expression, respectively, at 6 hrs following sonication reported here, it is plausible that there may be a causal link between the two observations^59^,^60^. While these transporters are largely expressed in brain microvessel endothelial cells, changes in their expression have been reported in astrocytes and other parenchymal cells following the induction of seizures^61^,^62^ or in HIV-1 infection of the brain^55^,^56^. Thus, it is possible the changes detected in the current study are influenced by gene expression changes in cells beyond ECs.

The observed changes in microvascular gene expression following sonication may also indicate an increase in early angiogenic processes, as evidenced by increased expression of several angiogenesis-related genes, including Timp1, Egr3, Lgals3, Mmp9, and Itgb2, as well as positive enrichment scores for several angiogenesis-related GO terms. FUS, in the presence of MBs, has previously been shown to enhance angiogenesis in skeletal muscle, with an approximately 65% increase in arterioles per muscle fiber reported at 7 and 14 days following sonication^63^. Similarly, microarray analysis of gene expression in ECs subjected to shear stress suggests that angiogenic pathways are upregulated after 24 hrs^64^. Given the well-established link between angiogenesis and inflammatory mediators^65^, as well as the results presented here, further investigation into the effects of FUS on blood vessel density and cerebral blood flow are warranted. If FUS can be shown to stimulate angiogenesis in the brain, this could have major implications for patients recovering from stroke or traumatic brain injury^66^ by providing a means to encourage the repair and regeneration of injured brain tissue and promote functional recovery.

In addition to increasing the permeability of the BBB, FUS has been reported to have a variety of other bioeffects in the CNS. The transcriptome data presented here may provide insight into the factors which drive these effects. We hypothesize that the stresses exerted on the microvascular walls by oscillating MBs at the focus of the ultrasound beam initiate an acute, transient, inflammatory response in vascular endothelium. This leads to an increase in proinflammatory cytokine and chemokine gene expression, contributing to many of the bioeffects reported to accompany FUS. Besides the potential influence on neurogenesis, angiogenesis, and transporter expression discussed above, acute inflammation could plausibly contribute to the increased endocytosis^3^, reduced immunoreactivity of tight junction proteins^4^, and reduction in Aβ load^11^,^12^,^13^ reported to follow FUS.

Electron microscopy evidence from Sheikov *et al*. demonstrated increases in the number of endocytotic vesicles in vascular ECs following FUS^3^. This observation is supported at the protein expression level with increases in the immunodetection of caveolin-1 post-sonication^26^. The rate of transcytosis across cerebral capillary ECs has previously been shown to be influenced by proinflammatory cytokines^67^, thus, it is possible that the changes in proinflammatory cytokine gene expression reported here may be a contributing factor to the increased EC endocytosis previously observed following FUS. Likewise, Sheikov *et al*. have also shown that immunodetection of tight junction proteins in EC clefts is reduced up to 4 hrs following FUS^4^. This effect may also be influenced by increased proinflammatory cytokine gene expression, as Ccl2 has been shown to mediate internalization of occludin and claudin-5 in brain ECs^68^.

Notably, FUS has been shown to reduce Aβ plaque load and improve performance in a variety of behavioural tasks in two mouse models of AD^11^,^12^,^13^. While there is ample evidence implicating neuroinflammation in the pathogenesis of AD^69^, it is possible that induction of a transient inflammatory response in brain microvessels following FUS could paradoxically contribute to an increase in plaque clearance. This could be achieved by the infiltration and activation of immune cells, such as Ccr2-positive mononuclear phagocytes^70^ and Ly6Clo monocytes^71^, that act to clear Aβ deposits. While much focus has been on reducing neuroinflammation to attenuate AD progression, it has been proposed that a rebalancing of innate immunity in the brain by inhibiting actions of key anti-inflammatory cytokines may encourage a reduction in AD pathology^72^. FUS may act to shift innate immunity in a similar way, contributing to the observed improvements in AD-like pathology in mice models.

As with any assessment of gene expression changes, there must be a level of caution taken when inferring conclusions regarding the functional outcomes of these changes since protein expression may not follow the same trend. However, the inclusion of GSEA may lessen this risk, as examining expression changes in groups of related genes acts to distribute the burden of conclusions across the entire genome rather than changes in single genes^73^. Moreover, while the cellular composition of samples used for microarray analysis can be characterized as greatly enriched with microvessels, they contain transcriptional markers of other cell types. Thus, some of the reported changes in gene expression may be influenced by cell types beyond the vasculature, especially astrocytes. Due to the unbiased method of collection, however, the cellular composition of samples should be consistent across groups. Additionally, the isolation of microvessels from brain tissue using any method results in some degree of cellular contamination^74^. Lastly, this study focused on the characterization of gene expression changes in microvasculature, as this is the site of increased permeability following FUS and experiences the largest magnitude of stress during sonication. Thus, the results presented here can not necessarily be extended to other tissue types within the brain or outside the CNS. Additionally, due to the specific focus on dorsal hippocampal microvessels in the present study, the transcriptional response to FUS in vasculature from other brain areas may not be consistent.

An assessment of gene expression changes can give great insight into the response of microvessels to FUS, however, the exploratory nature of this work necessitates further investigation. Among the most pertinent areas requiring research, determining the time course of the inflammatory response, as suggested by this work, is imperative. The gene expression profile in microvessels 24 hrs following sonication suggests that the inflammatory response is greatly dampened at this time point, however, given the acute nature of this investigation, a secondary rebound inflammatory response cannot be ruled out. In addition to investigating the long term impact of FUS, it will be important to look at how repeated sonications impact gene expression and functional changes in brain microvasculature. While previous long term survival and behavioural studies^23^ would suggest that there are no long term effects of FUS treatment, a full characterization of changes induced by any medical intervention is important from a safety perspective. Future work should also focus on determining if FUS can induce angiogenesis in the CNS, alters BBB transporter function, and whether the bioeffects reported to follow sonication are driven by the production of pro-inflammatory cytokines and chemokines.

The work presented here focused on characterizing acute gene expression changes in dorsal hippocampal microvessels following FUS-mediated increases in BBB permeability. While the exploratory nature of the work precludes drawing firm conclusions, the transcriptional changes observed suggest that there is a transient inflammatory response in microvessels that is greatly dampened by 24 hrs. GSEA and changes in the expression of individual genes would suggest that FUS may induce early angiogenic processes and reduce drug efflux across the BBB. Presented in the context of previous observations, the changes in gene expression reported here may suggest that many of the bioeffects of FUS are driven by the production of proinflammatory cytokines and chemokines.

The challenge of drug delivery to the CNS represents a substantial obstacle to the treatment of many neurological diseases. FUS has demonstrated great promise as a method to transiently increase the permeability of the BBB in a targeted manner and is starting to be tested in human trials. The safety profile of the technique appears high but further work is needed to fully characterize the long term effects of repeated FUS on healthy and pathological brain tissue. The opportunity for novel applications of FUS to be explored still exists, as the spectrum of its utility is yet to be fully identified. Efforts to apply FUS in the clinic should be strongly pursued due to its flexibility as a therapeutic intervention and the great need for new strategies in effective drug delivery and treatment of neurological disorders.

## Additional Information

**How to cite this article:** McMahon, D. *et al*. Acute effects of focused ultrasound-induced increases in blood-brain barrier permeability on rat microvascular transcriptome. *Sci. Rep.* **7**, 45657; doi: 10.1038/srep45657 (2017).

**Publisher's note:** Springer Nature remains neutral with regard to jurisdictional claims in published maps and institutional affiliations.

## Supplementary Material

Supplementary Information

## Figures and Tables

**Figure 6:**
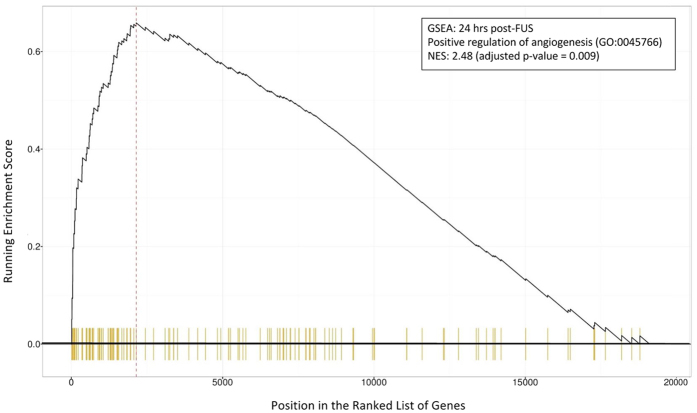
GSEA for positive regulation of angiogenesis 24 hrs post-FUS. The running enrichment score for the GO term “positive regulation of angiogenesis” (GO:0045766) is depicted in relation to a list of genes ranked by log2 fold change at 24 hrs post-FUS. Each vertical yellow line indicates the location of a gene in the ranked list that is associated with this GO term. NES = 2.48 (adjusted p = 0.009). GO term are comprised of a list of genes whose function are related. Thus, the non-random distribution of these genes within the ranked list is indicative of an activation of angiogenic processes.

**Table 1:** Primers used for microarray validation.

Target Gene	Fwd Sequence	Rev Sequence	Annealing Temperature (°C)
Gapdh	CAGGGCTGCCTTCTCTTGTG	GATGGTGATGGGTTTCCCGT	62.7
Cd74	AGCGCCCGTGAAGAATGTTA	CTGTGGGTAGTTCACGGGTC	61.1
Lcn2	GATTCGTCAGCTTTGCCAAGT	CATTGGTCGGTGGGAACAG	61.1
Slc22a6	CATTGCAATCAACTGCATGACACTA	AGGAACTGGCCCAGGCTGTA	62.7
Gfap	TGGCCACCAGTAACATGCAA	CAGTTGGCGGCGATAGTCAT	61.1
Abcb1a	TACATCTTGGCGGACCTTAC	CGCTGGTTTCTTTTCTTTCTTC	61.1
Itgb2	CAGCTGGCCCACAAACTTTC	TGGAATCGTCAGACAGCTCG	61.1
Ccl2	CCAGAAACCAGCCAACTCTC	GCTACAGGCAGCAACTGTGA	61.1
Serpine1	GAGGATGAAAGAAACAGCCAGCT	CCCGCTATGAAATTAGATTCACGT	61.4
Mmp9	TGCTCCTGGCTCTAGGCTAC	GCTTCTCTCCCATCATCTGG	61.4

**Table 2:** Differential expression of selected genes in hippocampal microvessels at 6 and 24 hrs post-sonication relative to contralateral hemisphere.

Related Functions	Entrez ID	Gene Symbol	6 hrs Post-FUS		24 hrs Post-FUS	
			Log2 FC	Adjusted P-Value	Log2 FC	Adjusted P-Value
Inflammation	24232	C3	**1.26**	**0.031**	**1.55**	**0.023**
	24770	Ccl2	**4.73**	**<0.001**	1.70	0.083
	25542	Ccl3	**1.83**	**0.005**	0.44	0.495
	287910	Ccl6	0.68	0.454	**1.94**	**0.045**
	287561	Ccl7	**3.57**	**0.005**	1.56	0.170
	60463	Ccr2	2.42	0.106	**4.11**	**0.023**
	117029	Ccr5	0.62	0.191	**1.15**	**0.036**
	81503	Cxcl1	**2.74**	**0.003**	0.47	0.622
	305236	Cxcl11	**2.25**	**0.003**	1.07	0.097
	24387	Gfap	0.29	0.468	**1.54**	**0.003**
	25712	Ifng	−0.20	0.374	−0.05	0.875
	24494	Il1b	**3.18**	**0.003**	0.35	0.762
	24498	Il6	**2.85**	**0.016**	1.78	0.119
	309684	Itgb2	0.47	0.427	**1.38**	**0.034**
	29527	Ptgs2	**0.65**	**0.049**	0.43	0.210
	25544	Sele	**3.82**	**<0.001**	0.40	0.650
	25651	Selp	1.83	0.076	0.38	0.788
	24835	Tnf	**0.90**	**0.037**	0.34	0.478
Oxidative Stress	24404	Gpx1	−0.27	0.399	0.59	0.077
	297029	Gstk1	−0.36	0.197	0.03	0.942
	24426	Gstp1	−0.25	0.415	0.00	0.993
	29253	Maoa	−0.28	0.462	0.19	0.663
	24598	Nos1	−0.40	0.515	0.10	0.914
	24599	Nos2	0.83	0.064	−0.07	0.925
	24600	Nos3	−0.17	0.803	0.42	0.484
	24786	Sod1	−0.02	0.831	−0.01	0.936
	24787	Sod2	**1.31**	**0.010**	0.28	0.623
	25352	Sod3	−0.44	0.066	0.43	0.087
Resolution of Inflammation	287454	Alox12	−0.12	0.760	0.00	0.997
	81639	Alox15	0.32	0.287	0.02	0.973
	25290	Alox5	−0.12	0.764	0.22	0.566
	29624	Alox5ap	−0.21	0.705	**1.13**	**0.033**
	79242	Hpgd	−0.41	0.569	0.81	0.235
	299732	Lta4h	−0.11	0.751	0.55	0.068
	59264	Ltb4r	−0.01	0.988	−0.05	0.887
	25211	Lyz2	0.79	0.321	**1.77**	**0.044**
	25526	Ptgds	−0.59	0.028	0.03	0.935
	81752	Ptger2	0.14	0.790	0.22	0.671
	59103	Ptges	**2.03**	**0.005**	0.82	0.216
	192227	Ptgr1	−1.07	0.065	0.15	0.865
	29527	Ptgs2	0.65	0.049	0.43	0.210
	364049	Slamf7	−0.20	0.708	**1.08**	**0.036**
	116510	Timp1	1.27	0.059	**2.59**	**0.003**
Abc and Slc Transporters	287788	Abca9	−**1.38**	**0.002**	0.26	0.548
	170913	Abcb1a	**−1.14**	**0.042**	0.08	0.927
	24646	Abcb1b	0.71	0.352	0.19	0.868
	24565	Abcc1	−0.05	0.903	0.23	0.522
	25303	Abcc2	0.00	0.995	−0.16	0.613
	170924	Abcc4	−0.69	0.180	0.10	0.901
	312382	Abcg2	−1.23	0.161	0.14	0.922
	64846	Slc13a3	−**1.31**	**0.043**	0.48	0.518
	503568	Slc13a4	−**1.72**	**0.002**	0.07	0.914
	246239	Slc15a3	**1.24**	**0.018**	0.82	0.107
	287450	Slc16a11	−**1.14**	**0.021**	−0.13	0.847
	295356	Slc16a4	−**1.11**	**0.015**	0.27	0.602
	29509	Slc22a6	**−1.83**	**0.003**	0.18	0.794
	83500	Slc22a8	**−1.19**	**0.028**	0.00	0.997
	366568	Slc30a3	**1.23**	**0.049**	−1.03	0.106
	170840	Slc40a1	−**1.42**	**0.028**	0.10	0.913
	171163	Slc6a13	**−1.85**	**0.001**	0.24	0.671
	499587	Slc7a14	**1.19**	**0.046**	−1.05	0.088
	170698	Slco1a2	−**1.13**	**0.037**	0.29	0.659
Angiogenesis	89807	Angpt1	−0.62	0.135	0.37	0.435
	89805	Angpt2	−0.23	0.648	0.76	0.091
	25148	Egr3	**1.23**	**0.005**	−0.42	0.294
	79114	Fgfr1	−0.14	0.576	0.23	0.351
	309684	Itgb2	0.47	0.427	**1.38**	**0.034**
	83781	Lgals3	0.42	0.527	**2.29**	**0.004**
	81687	Mmp9	**2.53**	**0.006**	1.55	0.083
	24628	Pdgfb	−0.07	0.893	0.18	0.699
	59086	Tgfb1	0.03	0.945	0.69	0.052
	83785	Vegfa	0.17	0.568	−0.1	0.798
Tight Junction Integrity	310655	Cgn	0.06	0.850	0.01	0.976
	65131	Cldn5	−0.75	0.069	0.09	0.885
	307505	Ctnna1	−0.39	0.168	0.31	0.310
	84353	Ctnnb1	−0.32	0.135	0.11	0.703
	619374	Jam2	−0.65	0.027	0.26	0.395
	83497	Ocln	−0.99	0.035	0.09	0.901
	292994	Tjp1	−0.42	0.073	0.12	0.696
	115769	Tjp2	−0.09	0.811	0.47	0.107
	314640	Tjp3	0.17	0.538	−0.07	0.853
Endocytosis	64310	Arf1	0.15	0.375	0.12	0.516
	79121	Arf6	−0.02	0.944	0.22	0.284
	25404	Cav1	−0.62	0.136	0.24	0.639
	64465	Cdc42	−0.16	0.101	0.07	0.493
	140694	Dnm1	0.56	0.111	−0.79	0.048
	313474	Eps15	−0.18	0.451	0.09	0.783
	64665	Flot1	−0.04	0.887	0.21	0.280
	83764	Flot2	0.07	0.818	0.16	0.554
	25150	Fyn	0.39	0.075	0.22	0.352
	287710	Ptrf	−0.61	0.104	0.19	0.688

**Table 3:** GSEA of selected GO terms in hippocampal microvessels at 6 and 24 hrs post-sonication relative to contralateral hemisphere

GO ID	GO Description	6 hrs Post-FUS		24 hrs Post-FUS	
		NES	Adjusted P-Value	NES	Adjusted P-Value
GO:0002544	chronic inflammatory response	**2.27**	**0.015**	**1.99**	**0.009**
GO:0002437	inflammatory response to antigenic stimulus	**2.18**	**0.015**	**2.13**	**0.009**
GO:0048514	blood vessel morphogenesis	−**1.57**	**0.015**	**2.34**	**0.009**
GO:0045766	positive regulation of angiogenesis	1.43	0.080	**2.48**	**0.009**
GO:0043536	positive regulation of blood vessel endothelial cell migration	−0.94	0.710	**1.95**	**0.009**
GO:1904018	positive regulation of vasculature development	1.39	0.101	**2.48**	**0.009**
GO:0010573	vascular endothelial growth factor production	**2.29**	**0.015**	**2.23**	**0.009**
GO:0022804	active transmembrane transporter activity	−**1.63**	**0.025**	−1.33	0.041
GO:0015171	amino acid transmembrane transporter activity	−**1.59**	**0.037**	−1.48	0.080
GO:0090484	drug transporter activity	**−1.85**	**0.037**	0.93	0.754
GO:0008028	monocarboxylic acid transmembrane transporter activity	**−1.97**	**0.025**	1.06	0.592
GO:0015291	secondary active transmembrane transporter activity	**−1.65**	**0.025**	−1.34	0.065
GO:0015085	calcium ion transmembrane transporter activity	**1.68**	**0.025**	−**2.37**	**0.020**
GO:0015108	chloride transmembrane transporter activity	1.04	0.630	−**1.93**	**0.020**
GO:0015075	ion transmembrane transporter activity	1.37	0.025	−**2.14**	**0.022**
GO:0015079	potassium ion transmembrane transporter activity	**1.91**	**0.025**	**−2.44**	**0.020**
GO:0015081	sodium ion transmembrane transporter activity	1.20	0.330	**−1.93**	**0.020**
