# Supplementary material for: Acute effects of focused ultrasound-induced increases in blood-brain barrier permeability on rat microvascular transcriptome
Source: Sci Rep. 2017 Apr 4;7:45657. doi: 10.1038/srep45657 (PMC5379491; doi:10.1038/srep45657)
Supplement: Supplementary Information [file srep45657-s1.pdf]

Acute effects of focused ultrasound-induced increases in blood-brain barrier permeability on rat microvascular transcriptome

Dallan McMahon, Reina Bendayan, and Kullervo Hynynen

SUPPLEMENTARY TABLE, FIGURES, AND DATA

Supplementary Data 1: MIAME-compliant microarray data deposited in ArrayExpress. Accession number: E-MTAB-5251

Supplementary Figures:

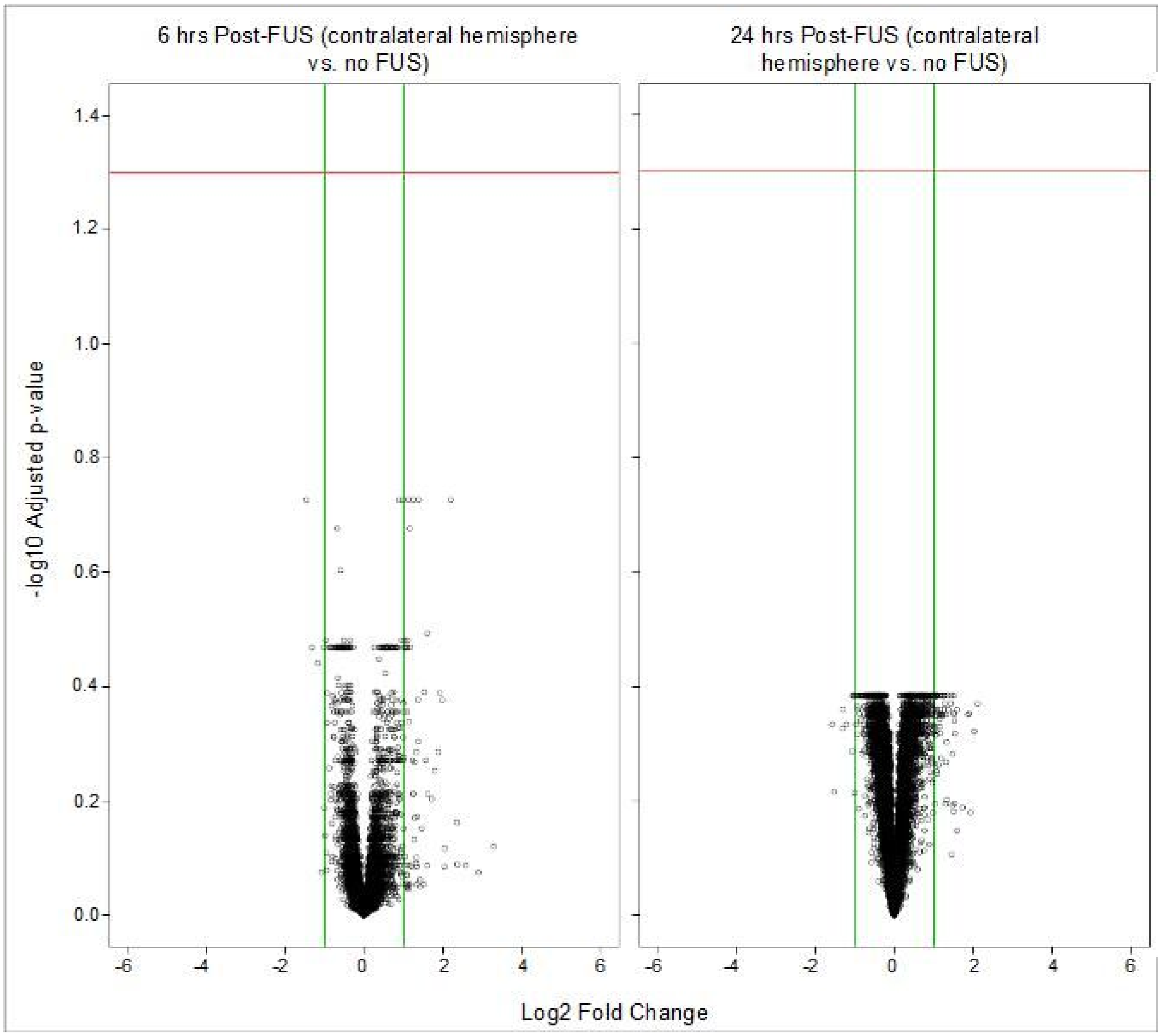

Supplementary Figure 1: Volcano plots of differential gene expression. Relative gene expression in hippocampal microvessels was compared between the hemispheres contralateral to sonication at 6 and 24 hrs post-FUS and animals that did not receive FUS. A positive log2 fold change indicates increased relative expression in microvessels contralateral to soniation compared to non-sonicated control animals. Green vertical lines indicate a log2 fold change of 1.0 or -1.0. Red horizontal lines indicate a -log10 adjusted p-value of 1.30, corresponding to an adjusted p-value of 0.05.

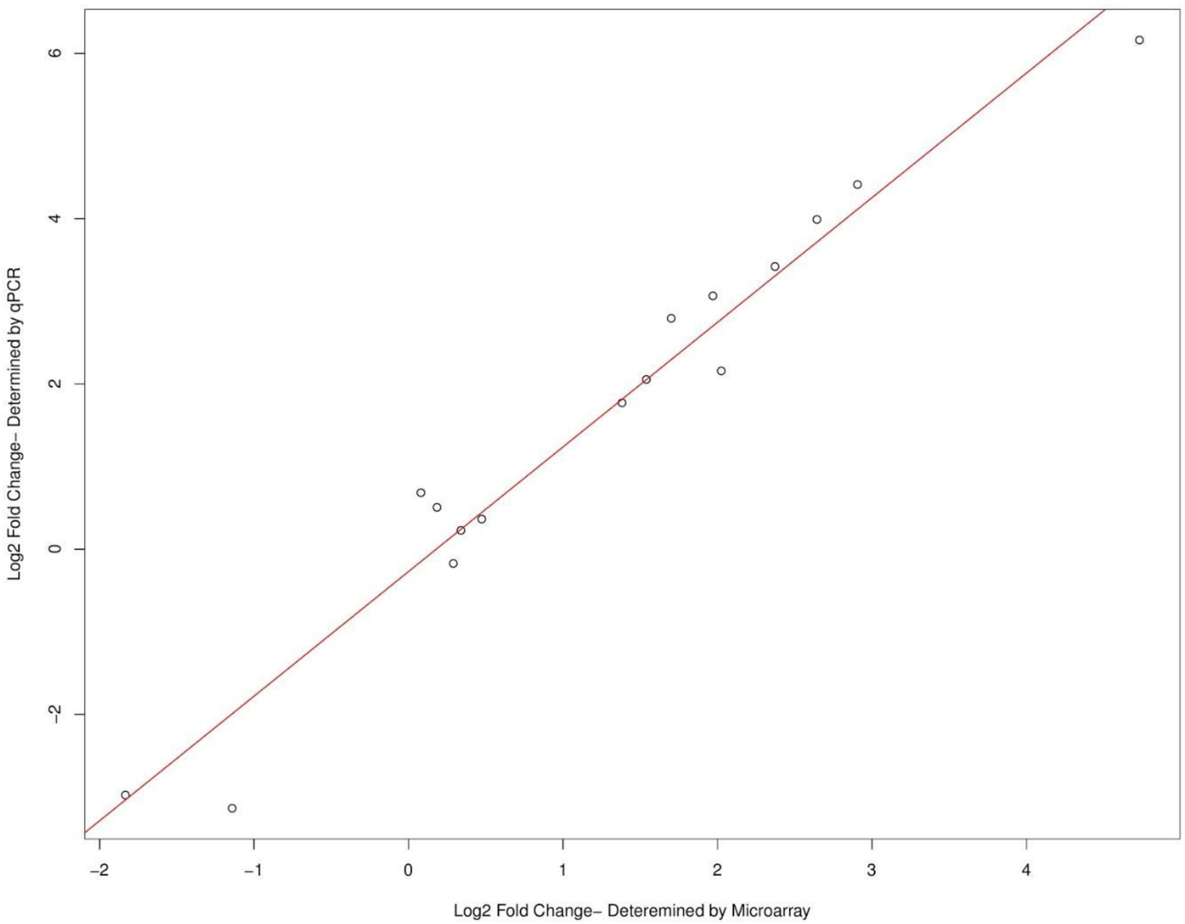

Supplementary Figure 2: Differential expression of selected genes at 6 and 24 hrs post-FUS as measured by microarray analysis and qRT-PCR. To validate differential gene expression results from the microarray analysis, qRT-PCR was used to assess relative expression of selected genes. A comparison of log2 fold changes at 6 and 24 hrs post-FUS (compared to the contralateral hemisphere), as determined by each of these methods, shows a high correlation (r^2=0.959). The log2 fold change values used to generate this plot can be found in Supplementary Table 8.

Supplementary Table 1: Upregulated genes in hippocampal microvessels at either 6 or 24 hrs following sonication (versus contralateral hemisphere)

		6 hrs Post-FUS		24 hrs Post-FUS	
**Entrez ID**	**Gene Symbol**	**Log2 FC**	**Adjusted P-Value**	**Log2 FC**	**Adjusted P-Value**
24153	A2m	0.37	0.585	1.73	0.019
300475	Adamts8	1.60	0.025	0.41	0.619
29624	Alox5ap	-0.21	0.705	1.13	0.032
54323	Arc	1.39	0.002	-0.42	0.273
498282	Arhgap30	0.30	0.546	1.01	0.043
497990	Arl5c	1.12	0.012	0.16	0.778
304648	Asf1b	-0.11	0.816	1.38	0.004
78971	Birc3	2.30	0.005	1.29	0.085
367901	Btk	-0.07	0.905	1.02	0.037
312705	C1r	-0.27	0.558	1.41	0.007
192262	C1s	0.10	0.874	1.69	0.004
24232	C3	1.26	0.031	1.55	0.023
84007	C3ar1	0.60	0.116	1.01	0.026
297339	Capg	-0.35	0.579	1.40	0.033
311327	Casc5	-0.09	0.897	1.17	0.034
24770	Ccl2	4.73	<0.001	1.70	0.083
25542	Ccl3	1.83	0.005	0.44	0.495

287910	Ccl6	0.68	0.454	1.94	0.045
287561	Ccl7	3.57	0.005	1.56	0.170
114494	Ccna2	-0.17	0.760	1.00	0.043
60463	Ccr2	2.42	0.106	4.11	0.023
117029	Ccr5	0.62	0.191	1.15	0.036
60350	Cd14	1.20	0.030	1.17	0.048
29185	Cd37	0.31	0.496	1.10	0.026
25406	Cd44	0.75	0.102	1.11	0.036
287435	Cd68	-0.03	0.971	1.52	0.006
303747	Cd7	0.07	0.939	1.54	0.031
25599	Cd74	0.34	0.732	2.64	0.009
366065	Cers6	1.12	0.006	-0.34	0.400
680338	Clec12a	-0.61	0.505	2.25	0.024
474143	Clec4a	-0.45	0.284	1.15	0.023
297584	Clec4a2	0.10	0.945	2.12	0.046
362431	Clec4a3	-0.68	0.272	1.87	0.012
502902	Clec7a	0.93	0.491	3.01	0.036
406864	Clic1	0.59	0.233	1.59	0.011
155151	Coro1a	1.00	0.040	0.63	0.205
24268	Cp	-0.01	0.990	1.86	0.002
24273	Cryaa	1.12	0.053	1.28	0.045
171081	Csf2rb	2.38	0.025	1.55	0.140
252929	Ctsz	0.25	0.609	1.37	0.012
89808	Cx3cl1	1.07	0.002	-0.51	0.089
81503	Cxcl1	2.74	0.003	0.47	0.621
305236	Cxcl11	2.25	0.003	1.07	0.097
497942	Cxcl16	0.70	0.201	1.31	0.039
66021	Cybb	-0.26	0.720	1.40	0.040
170901	Efna3	1.13	0.018	-0.40	0.421
25148	Egr3	1.23	0.005	-0.42	0.294
81505	Emp3	-0.15	0.818	1.24	0.031
295279	Fcgr1a	0.01	0.988	1.16	0.026
304966	Fcgr3a	0.08	0.936	1.44	0.048
114091	Fcnb	2.81	0.008	0.84	0.440
362332	Flnc	-0.17	0.761	1.08	0.039
314322	Fos	1.31	0.046	0.62	0.376
25445	Fosl1	2.06	0.006	0.51	0.517
499537	Fyb	0.52	0.257	1.09	0.036
171164	Gbp2	1.15	0.032	0.32	0.611
24387	Gfap	0.29	0.468	1.54	0.002
499914	Gins1	-0.28	0.739	2.04	0.012
299783	Glipr1	1.66	0.194	2.90	0.045
679819	Glipr2	0.64	0.161	1.40	0.012
113940	Gmfg	0.17	0.770	1.12	0.033
690825	Gngt2	0.28	0.775	1.78	0.041
113955	Gpnmb	-0.28	0.571	1.69	0.003
24409	Grin2a	1.03	0.026	-0.77	0.092
311984	Gsap	0.70	0.159	1.16	0.041
502125	Hist1h2ah	0.00	0.996	1.00	0.042
24471	Hspb1	0.17	0.785	1.56	0.012
306564	Htra4	1.47	0.005	0.14	0.823
293618	Ifitm1	-0.13	0.838	1.50	0.011
287813	Igsf7	0.78	0.424	2.18	0.039
24494	Il1b	3.18	0.003	0.35	0.762
24498	Il6	2.85	0.016	1.78	0.119
368066	Inmt	0.40	0.596	1.65	0.032
292060	Irf8	0.61	0.185	1.07	0.043
290651	Isyna1	-0.62	0.054	1.07	0.007
25021	Itgam	0.66	0.121	1.55	0.005
309684	Itgb2	0.47	0.427	1.38	0.034
24517	Junb	1.17	0.027	0.81	0.118
297666	Klra5	-0.25	0.657	1.85	0.003
317676	Lat2	0.08	0.885	1.22	0.011
170496	Lcn2	2.02	0.008	2.91	0.002
306071	Lcp1	0.36	0.558	1.37	0.034
83781	Lgals3	0.42	0.527	2.29	0.004

60584	Lif	1.13	0.018	-0.02	0.984
292594	Lilrb4	1.76	0.088	3.23	0.011
315691	Lingo1	1.37	0.013	-0.86	0.104
361680	Lsp1	1.05	0.183	2.08	0.026
291359	Ly86	-0.12	0.836	1.40	0.012
25211	Lyz2	0.79	0.321	1.77	0.044
291885	Mcm5	0.20	0.633	1.10	0.012
29685	Mcm6	0.17	0.682	1.01	0.019
291234	Mki67	-0.35	0.584	1.83	0.011
81687	Mmp9	2.53	0.006	1.55	0.083
304988	Mnda	0.55	0.521	1.75	0.045
361735	Ms4a6a	0.20	0.801	1.46	0.036
293749	Ms4a6bl	-0.86	0.091	1.43	0.020
314654	Myo1f	0.00	0.997	1.14	0.036
58982	Ncan	0.19	0.656	1.51	0.002
309452	Nfkb2	1.21	0.046	0.40	0.571
266777	Nptx1	1.32	0.028	-0.89	0.136
288475	Nptx2	1.55	<0.001	-0.40	0.234
83834	Nrn1	1.23	0.026	-0.99	0.078
24609	Odc1	1.03	0.023	0.70	0.112
289247	Olr1584	0.37	0.508	1.11	0.050
310132	Osmr	1.33	0.048	1.06	0.123
363041	Pate4	1.61	0.008	1.08	0.071
54320	Pdpn	0.40	0.429	1.23	0.031
85311	Pla1a	1.17	0.038	1.56	0.019
297694	Plbd1	-0.44	0.379	1.19	0.034
298199	Plin2	0.26	0.561	1.13	0.018
302562	Plp2	-0.06	0.938	1.29	0.032
59103	Ptges	2.03	0.005	0.82	0.216
362524	Ptpn3	1.02	0.045	-0.55	0.298
116689	Ptpn6	-0.05	0.939	1.11	0.034
689388	Ptx3	3.12	0.005	1.24	0.235
282817	Pycard	-0.06	0.943	1.36	0.025
365042	Rab32	0.92	0.150	1.42	0.050
366957	Rac2	0.82	0.158	1.65	0.020
364190	Rasl10a	1.11	0.026	-0.37	0.492
100360982	Relb	1.30	0.034	0.55	0.403
360857	Rgs16	1.43	0.050	0.90	0.229
289076	Rgs18	0.01	0.991	1.57	0.026
362993	Rnd1	1.65	0.013	-0.57	0.410
309621	RT1-Ba	0.44	0.629	2.41	0.012
309622	RT1-Bb	0.21	0.852	2.53	0.014
294269	RT1-Da	0.47	0.683	2.91	0.014
294273	RT1-DMb	0.01	0.986	1.05	0.025
81778	S100a10	0.31	0.647	1.28	0.046
445415	S100a11	-0.35	0.626	1.45	0.038
24615	S100a4	-0.24	0.732	1.62	0.020
25544	Sele	3.82	<0.001	0.40	0.650
313057	Serinc2	1.66	0.004	-0.34	0.560
24795	Serpina3n	0.98	0.212	1.86	0.039
24617	Serpine1	2.37	0.036	1.97	0.089
155183	Skap2	-0.07	0.896	1.01	0.023
364049	Slamf7	-0.20	0.708	1.08	0.035
289235	Slamf9	-0.58	0.238	1.11	0.047
246239	Slc15a3	1.24	0.018	0.82	0.107
366568	Slc30a3	1.23	0.049	-1.03	0.106
499587	Slc7a14	1.19	0.046	-1.05	0.088
303378	Slfn13	-0.18	0.756	1.23	0.025
24787	Sod2	1.31	0.009	0.28	0.623
294043	Sorcs3	1.07	0.025	-0.31	0.568
308341	Ssc5d	-0.35	0.310	1.02	0.014
292483	Stx11	1.17	0.035	-0.06	0.950
24886	Tbxas1	-0.08	0.912	1.10	0.049
116510	Timp1	1.27	0.059	2.59	0.003
24834	Tk1	-0.30	0.312	1.02	0.006
684440	Tlr8	-0.64	0.307	1.51	0.034

316516	Tmbim1	0.41	0.395	1.37	0.014
299339	Tnfaip2	2.14	0.005	0.52	0.495
683206	Tnfaip3	1.03	0.034	0.07	0.923
686008	Tnfrsf22	1.09	0.042	0.23	0.738
500590	Tnfrsf9	1.06	0.007	0.24	0.579
301229	Trem1	2.10	0.037	1.50	0.140
78969	Trib1	1.06	0.005	0.46	0.176
307351	Tubb6	0.65	0.232	1.53	0.021
361537	Tyrobp	0.36	0.393	1.17	0.018

Supplementary Table 2: Downregulated genes in hippocampal microvessels at either 6 or 24 hrs following sonication (versus contralateral hemisphere)

		6 hrs Post-FUS		24 hrs Post-FUS	
**Entrez ID**	**Gene Symbol**	**Log2 FC**	**Adjusted P-Value**	**Log2 FC**	**Adjusted P-Value**
287788	Abca9	-1.38	0.002	0.26	0.548
170913	Abcb1a	-1.14	0.042	0.08	0.927
314800	Acss3	-1.07	0.028	0.23	0.700
100363275	Adgra2	-1.07	0.025	0.38	0.443
24172	Adh1	-1.30	0.029	0.60	0.335
116676	Aldh1a2	-1.20	0.006	0.76	0.070
171100	Angptl2	-1.22	0.034	0.90	0.119
81641	Anpep	-1.03	0.007	0.52	0.151
29473	Aoc3	-1.31	0.021	0.32	0.616
306805	Aspn	-1.83	0.012	0.18	0.854
59302	Bmp15	-1.04	0.013	-0.30	0.497
85272	Bmp7	-1.33	0.004	0.69	0.092
140724	Cacng3	1.07	0.109	-1.55	0.040
24932	Cd4	-1.04	0.024	0.58	0.195
503009	Cdkl4	0.18	0.790	-1.34	0.034
365871	Ciart	-1.01	0.048	0.29	0.634
245978	Cklf	-1.32	0.021	0.66	0.240
294141	Clic2	-1.35	0.034	0.29	0.717
310201	Cmbl	-1.16	0.026	-0.06	0.946
114020	Cml5	-1.56	0.014	-0.19	0.819
84352	Col1a2	-1.01	0.006	0.48	0.164
361289	Colec12	-1.01	0.006	0.30	0.419
360611	Copz2	-1.06	0.003	0.71	0.039
500046	Cped1	-1.46	0.009	0.16	0.818
83575	Cpz	-1.23	0.002	0.26	0.475
498392	Cytl1	-1.09	0.032	-0.06	0.937
366270	Edn3	-1.32	0.009	-0.09	0.905
365691	Egflam	-1.12	0.025	0.16	0.806
25043	Eln	-1.11	0.031	0.31	0.610
85496	Enpp1	-1.09	0.013	0.35	0.437
25315	Ephx1	-1.30	0.003	0.15	0.762
156826	Eya2	-1.02	0.013	0.88	0.040
362336	Fam180a	-2.20	0.001	0.53	0.352
691221	Faxdc2	-1.02	0.039	0.22	0.726
29558	Fcgrt	-1.26	0.006	0.39	0.381
499856	Fibin	-1.07	0.015	0.27	0.590
362366	Fkbp14	-1.14	0.009	0.36	0.407
297123	Fkbp9	-1.15	0.013	0.49	0.276
25256	Fmo1	-1.46	0.006	0.27	0.645
84493	Fmo3	-1.38	0.033	0.46	0.522
64507	Fmod	-1.26	0.007	0.44	0.335
79209	Frk	-1.93	0.009	0.16	0.872
24947	Gabra3	0.75	0.211	-1.34	0.048
394266	Gjb2	-1.58	0.004	0.13	0.853
84403	Gjb6	-1.04	0.048	-0.48	0.395
25236	Gpc3	-1.28	0.011	0.45	0.376
60667	Gpr20	-1.06	0.042	-0.06	0.942
300850	Gsta4	-1.20	0.014	0.04	0.958
24424	Gstm2	-1.70	0.006	0.21	0.776
362540	Hacd4	-1.03	0.049	0.46	0.426

24450	Hmgcs2	-1.08	0.028	-0.31	0.578
100359865	Hrct1	-1.08	0.049	0.16	0.834
161476	Hspb2	-1.09	0.007	0.48	0.212
686539	Islr	-1.31	0.009	0.80	0.099
25118	Itga1	-1.02	0.042	0.23	0.723
364786	Itga8	-1.09	0.038	0.13	0.866
100361376	Kank2	-1.04	0.012	0.15	0.769
316758	Lama1	-1.11	0.016	0.44	0.334
361303	Lims2	-1.06	0.032	0.13	0.853
100361383	LOC100361383	-1.07	0.040	-0.02	0.978
685513	LOC685513	-1.04	0.038	0.80	0.113
688126	LOC688126	-1.15	0.030	0.06	0.938
315714	Loxl1	-1.04	0.006	0.49	0.160
81682	Lum	-1.18	0.027	0.64	0.234
304131	Map3k7cl	-1.28	0.030	0.31	0.666
292671	Mill1	-1.67	0.006	0.19	0.796
300679	Mpzl2	-1.48	0.026	0.28	0.737
498011	Mrc2	-1.02	0.009	0.37	0.336
404641	Mrgprh	-1.49	0.028	0.06	0.959
361734	Ms4a4a	-1.36	0.039	0.88	0.188
313770	Mxra8	-1.19	0.006	0.40	0.333
24582	Myh11	-1.11	0.042	0.36	0.579
64570	Nat8	-1.47	0.009	0.16	0.829
312401	Ndnf	-1.63	0.003	0.18	0.764
246172	Nexn	-1.01	0.029	0.16	0.792
100912108	Nupr1	-1.15	0.028	0.80	0.120
500992	Nxpe1	-1.51	0.026	0.03	0.981
304507	Oas1i	-1.20	0.042	0.53	0.410
291015	Ogn	-1.04	0.004	0.11	0.803
83717	Omd	-1.75	0.002	-0.01	0.984
29569	Pcolce	-1.05	0.007	0.45	0.227
89813	Pdk4	-1.48	0.005	0.03	0.974
685611	Phldb2	-1.07	0.023	0.12	0.853
64672	Pln	-1.59	0.005	0.52	0.337
84400	Prelp	-1.07	0.009	0.34	0.403
60357	Prom1	-1.26	0.042	-0.06	0.949
171452	Rab3il1	-1.04	0.005	0.56	0.098
294789	Ranbp3l	-1.39	0.013	0.78	0.145
361819	RGD1566085	-1.08	0.049	-0.13	0.870
297757	Sbspon	-1.01	0.050	0.34	0.578
140927	Selenbp1	-1.22	0.006	0.01	0.984
84402	Sfrp1	-1.34	0.013	0.12	0.879
64846	Slc13a3	-1.31	0.043	0.48	0.518
503568	Slc13a4	-1.72	0.002	0.07	0.914
287450	Slc16a11	-1.14	0.020	-0.13	0.847
295356	Slc16a4	-1.11	0.015	0.27	0.602
29509	Slc22a6	-1.83	0.003	0.18	0.794
83500	Slc22a8	-1.19	0.028	0.00	0.997
170840	Slc40a1	-1.42	0.028	0.10	0.913
171163	Slc6a13	-1.85	0.001	0.24	0.671
170698	Slco1a2	-1.13	0.037	0.29	0.659
306147	Slitrk1	0.62	0.332	-1.41	0.045
25554	Snai2	-1.03	0.018	0.47	0.267
498159	Spry3	0.61	0.260	-1.19	0.048
83783	Sult1a1	-1.56	0.008	-0.09	0.910
192189	Syt17	0.28	0.620	-1.16	0.037
292406	Thbs2	-1.04	0.003	0.38	0.231
245953	Tmem37	-1.26	0.007	0.59	0.182
293874	Trpm6	-1.27	0.025	0.62	0.275
117514	Txnip	-1.02	0.009	0.44	0.241
89818	Vamp5	-1.07	0.013	0.62	0.140
361954	Veph1	-1.01	0.005	0.37	0.267
24877	Vsnl1	0.54	0.240	-1.02	0.048
64566	Wnt5a	-1.08	0.013	0.05	0.934
361552	Wtip	-1.50	0.014	0.16	0.848

Supplementary Table 3: GO terms (molecular function) with significant positive or negative enrichment in GSEA at either 6 or 24 hrs post-FUS (versus contralateral hemisphere)

		6 hrs Post-FUS		24 hrs Post-FUS	
**GO ID**	**GO Description**	**NES**	**Adjusted P-Value**	**NES**	**Adjusted P-Value**
GO:0015464	acetylcholine receptor activity	1.5	0.194	-1.69	0.045
GO:0022804	active transmembrane transporter activity	-1.63	0.025	-1.33	0.041
GO:0003995	acyl-CoA dehydrogenase activity	-1.91	0.037		>0.99
GO:0008179	adenylate cyclase binding	1.21	0.488	-1.69	0.045
GO:0031420	alkali metal ion binding	1.64	0.094	-1.97	0.02
GO:0033218	amide binding	-1.37	0.094	1.58	0.022
GO:0043176	amine binding	1.21	0.488	-1.76	0.02
GO:0015171	amino acid transmembrane transporter activity	-1.59	0.037	-1.48	0.08
GO:0004177	aminopeptidase activity	-1.7	0.069	1.91	0.02
GO:0070405	ammonium ion binding	1.56	0.111	-1.82	0.02
GO:0005253	anion channel activity	1.07	0.577	-1.97	0.02
GO:0008509	anion transmembrane transporter activity	-1.65	0.025	-1.71	0.02
GO:0015296	anion:cation symporter activity	-1.41	0.193	-1.55	0.045
GO:0003823	antigen binding	-1.07	0.582	2.24	0.02
GO:0016209	antioxidant activity	1.52	0.125	1.77	0.02
GO:0004003	ATP-dependent DNA helicase activity	-0.71	0.966	1.74	0.045
GO:0042625	ATPase activity, coupled to transmembrane movement of ions	1.01	0.669	-1.57	0.046
GO:0008503	benzodiazepine receptor activity	1.46	0.236	-1.79	0.037
GO:0008013	beta-catenin binding	-1.64	0.025		>0.99
GO:0015106	bicarbonate transmembrane transporter activity	1.08	0.606	-1.85	0.02
GO:0016493	C-C chemokine receptor activity	1.87	0.025	1.69	0.056
GO:0005262	calcium channel activity	1.7	0.025	-2.26	0.02
GO:0005246	calcium channel regulator activity	1.44	0.2	-2.15	0.02
GO:0015085	calcium ion transmembrane transporter activity	1.68	0.025	-2.37	0.02
GO:0004683	calmodulin-dependent protein kinase activity	1.33	0.282	-1.84	0.029
GO:0005516	calmodulin binding	1.66	0.025	-2.23	0.021
GO:0030246	carbohydrate binding	1.25	0.159	2.05	0.02
GO:0016835	carbon-oxygen lyase activity	-1.72	0.025	-0.88	0.841
GO:0046943	carboxylic acid transmembrane transporter activity	-1.77	0.025	-1.33	0.144
GO:0004180	carboxypeptidase activity	-1.78	0.037	1.06	0.567
GO:0038024	cargo receptor activity	-1.76	0.046	1.95	0.02
GO:0005261	cation channel activity	1.95	0.025	-2.47	0.021
GO:0008324	cation transmembrane transporter activity	1.5	0.025	-2.11	0.022
GO:0048020	CCR chemokine receptor binding	2.55	0.025	2.2	0.02
GO:0050839	cell adhesion molecule binding	-1.29	0.183	1.8	0.02
GO:0015267	channel activity	1.83	0.025	-2.45	0.021
GO:0016247	channel regulator activity	1.56	0.047	-2.19	0.02
GO:0008009	chemokine activity	2.47	0.025	2.07	0.02
GO:0019956	chemokine binding	1.56	0.165	2.18	0.02
GO:0004950	chemokine receptor activity	1.58	0.154	1.96	0.02
GO:0042379	chemokine receptor binding	2.56	0.025	2.33	0.02
GO:0005254	chloride channel activity	1.09	0.554	-1.99	0.02
GO:0015108	chloride transmembrane transporter activity	1.04	0.63	-1.93	0.02
GO:0030276	clathrin binding	1.84	0.025	-2.21	0.02
GO:0050662	coenzyme binding	-1.79	0.025	1.35	0.063
GO:0048037	cofactor binding	-1.8	0.025	1.24	0.14
GO:0005518	collagen binding	-2.29	0.025	1.69	0.039
GO:0001848	complement binding	1.08	0.599	2.03	0.02
GO:0045236	CXCR chemokine receptor binding	1.82	0.037	1.8	0.025
GO:0005125	cytokine activity	2.19	0.025	2.08	0.02
GO:0019955	cytokine binding	1.44	0.117	2.46	0.02
GO:0004896	cytokine receptor activity	1.81	0.025	2.35	0.02
GO:0005126	cytokine receptor binding	1.97	0.025	2.28	0.02
GO:0005251	delayed rectifier potassium channel activity	2.08	0.025	-2.38	0.02
GO:0072509	divalent inorganic cation transmembrane transporter activity	1.64	0.025	-2.14	0.02
GO:0008094	DNA-dependent ATPase activity	-1.01	0.676	1.79	0.02
GO:0008301	DNA binding, bending	-1.82	0.037	1.52	0.122
GO:0003690	double-stranded DNA binding	0.97	0.741	1.52	0.049
GO:0015238	drug transmembrane transporter activity	-1.81	0.047	0.94	0.73

GO:0090484	drug transporter activity	-1.85	0.037	0.93	0.754
GO:0004866	endopeptidase inhibitor activity	1.46	0.047	2.16	0.02
GO:0061135	endopeptidase regulator activity	1.42	0.084	2.17	0.02
GO:0004857	enzyme inhibitor activity	1.1	0.423	1.84	0.02
GO:0046875	ephrin receptor binding	1.74	0.047	-1.3	0.321
GO:0005231	excitatory extracellular ligand-gated ion channel activity	1.82	0.025	-2.21	0.02
GO:0004527	exonuclease activity	-0.74	0.976	1.74	0.02
GO:0008238	exopeptidase activity	-1.91	0.025	1.23	0.318
GO:0005234	extracellular-glutamate-gated ion channel activity	1.96	0.025	-2.31	0.02
GO:0005230	extracellular ligand-gated ion channel activity	2.04	0.025	-2.57	0.02
GO:0050840	extracellular matrix binding	-1.56	0.106	2.17	0.02
GO:0005201	extracellular matrix structural constituent	-2.2	0.025	1.92	0.02
GO:0000062	fatty-acyl-CoA binding	-1.75	0.037	0.83	0.862
GO:0005504	fatty acid binding	1.35	0.268	1.66	0.039
GO:0001968	fibronectin binding	-1.12	0.561	1.83	0.02
GO:0050660	flavin adenine dinucleotide binding	-1.73	0.037	1.42	0.09
GO:0005109	frizzled binding	-1.87	0.025	0.72	0.973
GO:0008227	G-protein coupled amine receptor activity	1.71	0.056	-2.16	0.02
GO:0001637	G-protein coupled chemoattractant receptor activity	1.58	0.154	1.96	0.02
GO:0001664	G-protein coupled receptor binding	2	0.025	1.36	0.052
GO:0004890	GABA-A receptor activity	1.62	0.104	-2.21	0.02
GO:0016917	GABA receptor activity	1.75	0.066	-2.27	0.02
GO:0050811	GABA receptor binding	1.03	0.672	-1.81	0.037
GO:0022836	gated channel activity	2.16	0.025	-2.7	0.021
GO:0016595	glutamate binding	1.6	0.177	-2.05	0.02
GO:0008066	glutamate receptor activity	2.22	0.025	-2.49	0.02
GO:0035254	glutamate receptor binding	1.56	0.127	-2.05	0.02
GO:0004602	glutathione peroxidase activity	-1.97	0.025	1.5	0.138
GO:0004364	glutathione transferase activity	-1.87	0.037	0.79	0.91
GO:0001948	glycoprotein binding	-1.29	0.245	1.55	0.031
GO:0005539	glycosaminoglycan binding	1.25	0.217	2.13	0.02
GO:0019838	growth factor binding	-1.31	0.195	2.08	0.02
GO:0070851	growth factor receptor binding	1.52	0.056	1.72	0.02
GO:0003924	GTPase activity	1.72	0.038	1.16	0.339
GO:0008201	heparin binding	1.34	0.153	2.1	0.02
GO:0008331	high voltage-gated calcium channel activity	1.97	0.025	-1.97	0.02
GO:0005179	hormone activity	-0.96	0.753	-1.51	0.03
GO:0016836	hydro-lyase activity	-1.67	0.037	-1.1	0.5
GO:0016814	hydrolase activity, acting on carbon-nitrogen (but not peptide) bonds, in cyclic amidines	0.89	0.816	1.77	0.031
GO:0016798	hydrolase activity, acting on glycosyl bonds	-1.66	0.025	1.53	0.038
GO:0004553	hydrolase activity, hydrolyzing O-glycosyl compounds	-1.71	0.025	1.68	0.039
GO:0019865	immunoglobulin binding	-1.34	0.306	2.26	0.02
GO:0005237	inhibitory extracellular ligand-gated ion channel activity	1.49	0.231	-2.1	0.02
GO:0015103	inorganic anion transmembrane transporter activity	-1.05	0.598	-1.95	0.02
GO:0022890	inorganic cation transmembrane transporter activity	1.65	0.025	-2.25	0.021
GO:0005178	integrin binding	-1.73	0.025	2.2	0.02
GO:0005149	interleukin-1 receptor binding	1.96	0.025	1.46	0.196
GO:0005242	inward rectifier potassium channel activity	1.29	0.381	-2.06	0.02
GO:0005216	ion channel activity	1.93	0.025	-2.54	0.021
GO:0044325	ion channel binding	1.84	0.025	-2.02	0.02
GO:0015075	ion transmembrane transporter activity	1.37	0.025	-2.14	0.022
GO:0004970	ionotropic glutamate receptor activity	1.95	0.025	-2.21	0.02
GO:0035255	ionotropic glutamate receptor binding	1.02	0.672	-1.75	0.039
GO:0016853	isomerase activity	-1.28	0.203	1.53	0.039
GO:0019840	isoprenoid binding	-1.8	0.037	1.3	0.26
GO:0019894	kinesin binding	2.09	0.025	-2.01	0.038
GO:0015179	L-amino acid transmembrane transporter activity	1.01	0.672	-1.85	0.02
GO:0005313	L-glutamate transmembrane transporter activity	0.87	0.817	-1.65	0.045
GO:0043236	laminin binding	-1.65	0.08	1.85	0.02
GO:0022840	leak channel activity	1.13	0.559	-1.76	0.027
GO:0022834	ligand-gated channel activity	1.83	0.025	-2.48	0.02
GO:0015276	ligand-gated ion channel activity	1.83	0.025	-2.48	0.02
GO:0016829	lyase activity	-1.62	0.025	0.84	0.944
GO:0044877	macromolecular complex binding	-1	0.661	1.6	0.02
GO:0033549	MAP kinase phosphatase activity	2.06	0.025	1.12	0.533

GO:0017017	MAP kinase tyrosine/serine/threonine phosphatase activity	2.03	0.025	1.13	0.526
GO:0046873	metal ion transmembrane transporter activity	1.78	0.025	-2.4	0.02
GO:0042288	MHC class I protein binding	0.69	0.971	1.83	0.024
GO:0023026	MHC class II protein complex binding	1.69	0.103	2.06	0.02
GO:0042287	MHC protein binding	1.15	0.517	1.93	0.02
GO:0023023	MHC protein complex binding	1.84	0.047	2	0.02
GO:0072349	modified amino acid transmembrane transporter activity	-1.83	0.047	1.54	0.127
GO:0033293	monocarboxylic acid binding	1.02	0.672	1.63	0.045
GO:0008028	monocarboxylic acid transmembrane transporter activity	-1.97	0.025	1.06	0.592
GO:0048029	monosaccharide binding	-1.22	0.381	1.77	0.022
GO:0015077	monovalent inorganic cation transmembrane transporter activity	1.55	0.025	-2.21	0.021
GO:0051287	NAD binding	-1.76	0.025	0.95	0.753
GO:0022842	narrow pore channel activity	1.13	0.559	-1.76	0.027
GO:0008188	neuropeptide receptor activity	1.77	0.048	-1.53	0.093
GO:0042165	neurotransmitter binding	1.5	0.194	-1.92	0.02
GO:0030594	neurotransmitter receptor activity	1.97	0.025	-2.19	0.02
GO:0004715	non-membrane spanning protein tyrosine kinase activity	-0.98	0.72	1.93	0.02
GO:0004518	nuclease activity	-0.91	0.855	1.62	0.02
GO:0031492	nucleosomal DNA binding	-1.69	0.037	1.47	0.152
GO:0031491	nucleosome binding	-1.51	0.123	1.71	0.023
GO:0005342	organic acid transmembrane transporter activity	-1.76	0.025	-1.32	0.143
GO:0008514	organic anion transmembrane transporter activity	-1.79	0.025	-1.47	0.04
GO:0016491	oxidoreductase activity	-1.71	0.025	1.44	0.02
GO:0016614	oxidoreductase activity, acting on CH-OH group of donors	-1.59	0.047	1.09	0.488
GO:0016684	oxidoreductase activity, acting on peroxide as acceptor	-1.48	0.168	1.61	0.045
GO:0016627	oxidoreductase activity, acting on the CH-CH group of donors	-2.12	0.025	0.89	0.823
GO:0016628	oxidoreductase activity, acting on the CH-CH group of donors, NAD or NADP as acceptor	-1.86	0.037	1.08	0.567
GO:0052890	oxidoreductase activity, acting on the CH-CH group of donors, with a flavin as acceptor	-1.8	0.047		>0.99
GO:0016616	oxidoreductase activity, acting on the CH-OH group of donors, NAD or NADP as acceptor	-1.65	0.025	1.13	0.398
GO:0022803	passive transmembrane transporter activity	1.83	0.025	-2.45	0.021
GO:0001871	pattern binding	-1.25	0.407	1.78	0.045
GO:0038187	pattern recognition receptor activity	1.35	0.286	2.02	0.02
GO:0030165	PDZ domain binding	1.18	0.356	-1.99	0.02
GO:0016504	peptidase activator activity	-1.88	0.025	1.75	0.045
GO:0030414	peptidase inhibitor activity	1.37	0.122	2.12	0.02
GO:0061134	peptidase regulator activity	-1.17	0.341	2.15	0.02
GO:0042605	peptide antigen binding	-1.27	0.365	2.17	0.02
GO:0042277	peptide binding	-1.27	0.187	1.61	0.022
GO:0004601	peroxidase activity	-1.47	0.168	1.61	0.039
GO:0043274	phospholipase binding	1.97	0.025	-1.12	0.514
GO:0016775	phosphotransferase activity, nitrogenous group as acceptor	1.77	0.037	-1.74	0.051
GO:0001784	phosphotyrosine binding	1.67	0.083	1.71	0.039
GO:0048407	platelet-derived growth factor binding	-1.92	0.025	1.68	0.072
GO:0030247	polysaccharide binding	-1.25	0.407	1.78	0.045
GO:0005267	potassium channel activity	2.03	0.025	-2.54	0.02
GO:0015459	potassium channel regulator activity	0.97	0.741	-1.54	0.045
GO:0022841	potassium ion leak channel activity	1.06	0.617	-1.75	0.037
GO:0015079	potassium ion transmembrane transporter activity	1.91	0.025	-2.44	0.02
GO:0002020	protease binding	1.28	0.245	2.14	0.02
GO:0032403	protein complex binding	-1.09	0.327	1.65	0.02
GO:1990782	protein tyrosine kinase binding	-1.42	0.162	1.62	0.045
GO:0005102	receptor binding	1.43	0.025	1.52	0.02
GO:0030971	receptor tyrosine kinase binding	-1.31	0.274	1.62	0.045
GO:0070491	repressing transcription factor binding	-1.13	0.513	1.58	0.024
GO:0005501	retinoid binding	-1.87	0.037	1.34	0.265
GO:0004745	retinol dehydrogenase activity	-1.46	0.231	1.69	0.046
GO:0003723	RNA binding	0.95	0.921	1.64	0.02
GO:0044548	S100 protein binding	-1.64	0.115	2.01	0.02
GO:0005044	scavenger receptor activity	-2.01	0.025	1.95	0.02
GO:0015291	secondary active transmembrane transporter activity	-1.65	0.025	-1.34	0.065
GO:0004867	serine-type endopeptidase inhibitor activity	1.31	0.268	2.06	0.02

GO:0004993	serotonin receptor activity	1.88	0.047	-2.03	0.02
GO:0008329	signaling pattern recognition receptor activity	1.35	0.286	2.02	0.02
GO:0003697	single-stranded DNA binding	-0.86	0.882	1.83	0.02
GO:0000149	SNARE binding	1.76	0.025	-2.19	0.02
GO:0030515	snoRNA binding	1.94	0.025	1.08	0.578
GO:0005272	sodium channel activity	2.13	0.025	-1.86	0.02
GO:0017080	sodium channel regulator activity	1.14	0.488	-1.67	0.027
GO:0031402	sodium ion binding	1.55	0.178	-1.96	0.025
GO:0015081	sodium ion transmembrane transporter activity	1.2	0.33	-1.93	0.02
GO:0015294	solute:cation symporter activity	-1.54	0.047	-1.32	0.172
GO:0030507	spectrin binding	1.65	0.115	-1.76	0.037
GO:0003735	structural constituent of ribosome	-1.23	0.232	2.4	0.02
GO:0005198	structural molecule activity	-1.05	0.503	1.82	0.02
GO:0043566	structure-specific DNA binding	-0.98	0.721	1.7	0.02
GO:0022838	substrate-specific channel activity	1.93	0.025	-2.52	0.021
GO:0022891	substrate-specific transmembrane transporter activity	1.38	0.025	-2.06	0.022
GO:0022892	substrate-specific transporter activity	1.34	0.025	-1.9	0.023
GO:1901681	sulfur compound binding	-1.45	0.047	1.89	0.02
GO:0015293	symporter activity	-1.54	0.047	-1.26	0.21
GO:0017075	syntaxin-1 binding	1.75	0.047	-2.08	0.02
GO:0019905	syntaxin binding	1.73	0.038	-2.21	0.02
GO:0050321	tau-protein kinase activity	1.44	0.251	-1.78	0.039
GO:0050431	transforming growth factor beta binding	-1.85	0.047	2.03	0.02
GO:0022857	transmembrane transporter activity	1.27	0.056	-2	0.023
GO:0005215	transporter activity	1.24	0.037	-1.79	0.025
GO:0015631	tubulin binding	1.55	0.025	-1.43	0.041
GO:0001618	virus receptor activity	-1.42	0.283	1.69	0.04
GO:0019842	vitamin binding	-2.22	0.025	1.16	0.388
GO:0005245	voltage-gated calcium channel activity	1.92	0.025	-2.56	0.02
GO:0022843	voltage-gated cation channel activity	2.41	0.025	-2.81	0.02
GO:0022832	voltage-gated channel activity	2.22	0.025	-2.72	0.02
GO:0005244	voltage-gated ion channel activity	2.22	0.025	-2.72	0.02
GO:0005249	voltage-gated potassium channel activity	2.12	0.025	-2.64	0.02
GO:0005248	voltage-gated sodium channel activity	2.04	0.025	-1.66	0.094
GO:0017147	Wnt-protein binding	-1.94	0.025	1.41	0.221

Supplementary Table 4: GO terms (biological process) with significant positive or negative enrichment in GSEA at either 6 or 24 hrs post-FUS (versus contralateral hemisphere)

		6 hrs Post-FUS		24 hrs Post-FUS	
**GO ID**	**GO Description**	**NES)**	**Adjusted P-Value**	**NES**	**Adjusted P-Value**
GO:0001508	action potential	1.86	0.015	-2.34	0.01
GO:0002253	activation of immune response	1.25	0.132	2.54	0.009
GO:0002218	activation of innate immune response	1.31	0.172	2.48	0.009
GO:0006953	acute-phase response	2.12	0.015	2.08	0.009
GO:0002526	acute inflammatory response	1.88	0.015	2.28	0.009
GO:0002250	adaptive immune response	1.52	0.024	2.59	0.009
GO:0002460	adaptive immune response based on somatic recombination of immune receptors built from immunoglobulin superfamily domains	1.67	0.015	2.58	0.009
GO:0030534	adult behavior	2.05	0.015	-2.51	0.01
GO:0008344	adult locomotory behavior	1.72	0.015	-2.25	0.01
GO:0007628	adult walking behavior	1.81	0.024	-2.06	0.01
GO:0046631	alpha-beta T cell activation	1.39	0.089	2.38	0.009
GO:0046632	alpha-beta T cell differentiation	1.22	0.285	2.06	0.009
GO:0046633	alpha-beta T cell proliferation	1.28	0.29	2.18	0.009
GO:0015837	amine transport	1.89	0.015	-2.08	0.01
GO:0001525	angiogenesis	-1.4	0.024	2.4	0.009
GO:0019882	antigen processing and presentation	1.12	0.44	2.7	0.009
GO:0019884	antigen processing and presentation of exogenous antigen	0.78	0.898	2.45	0.009
GO:0002478	antigen processing and presentation of exogenous peptide antigen	1.08	0.549	2.42	0.009
GO:0019886	antigen processing and presentation of exogenous peptide antigen via MHC class II	0.88	0.768	2.25	0.009

GO:0048002	antigen processing and presentation of peptide antigen	-1.06	0.542	2.61	0.009
GO:0002474	antigen processing and presentation of peptide antigen via MHC class I	-1.54	0.089	2.17	0.009
GO:0002495	antigen processing and presentation of peptide antigen via MHC class II	1.11	0.497	2.42	0.009
GO:0002504	antigen processing and presentation of peptide or polysaccharide antigen via MHC class II	1.33	0.279	2.53	0.009
GO:0035904	aorta development	-2.08	0.015	1.6	0.051
GO:0043277	apoptotic cell clearance	1.63	0.075	2.14	0.009
GO:0060840	artery development	-2.12	0.015	1.72	0.009
GO:0048844	artery morphogenesis	-2.05	0.015	1.77	0.009
GO:0008306	associative learning	1.99	0.015	-2.18	0.01
GO:0061564	axon development	1.85	0.015	-2.18	0.011
GO:0007411	axon guidance	1.46	0.048	-2.08	0.011
GO:0007413	axonal fasciculation	2.07	0.015	-2.15	0.01
GO:0007409	axonogenesis	1.89	0.015	-2.27	0.012
GO:0042113	B cell activation	1.15	0.305	2.17	0.009
GO:0001783	B cell apoptotic process	0.78	0.889	2.02	0.009
GO:0019724	B cell mediated immunity	1.26	0.24	2.5	0.009
GO:0042100	B cell proliferation	1.01	0.606	2.06	0.009
GO:0050853	B cell receptor signaling pathway	0.82	0.871	2	0.009
GO:0007610	behavior	2.09	0.015	-1.78	0.013
GO:0002209	behavioral defense response	1.48	0.115	-2.33	0.01
GO:0001662	behavioral fear response	1.44	0.132	-2.28	0.01
GO:0007596	blood coagulation	-1.23	0.203	2.21	0.009
GO:0001568	blood vessel development	-1.68	0.015	2.32	0.009
GO:0048514	blood vessel morphogenesis	-1.57	0.015	2.34	0.009
GO:0001974	blood vessel remodeling	-1.16	0.419	2	0.009
GO:0046849	bone remodeling	-1.31	0.139	2.05	0.009
GO:0017156	calcium ion-dependent exocytosis	2	0.015	-2.33	0.01
GO:0048791	calcium ion-dependent exocytosis of neurotransmitter	2.14	0.015	-2.51	0.01
GO:0070588	calcium ion transmembrane transport	1.43	0.054	-2.18	0.01
GO:0072358	cardiovascular system development	-1.68	0.015	2.04	0.009
GO:0050432	catecholamine secretion	1.59	0.055	-2.2	0.01
GO:0051937	catecholamine transport	1.52	0.076	-2.15	0.01
GO:0098655	cation transmembrane transport	1.62	0.015	-2.14	0.013
GO:0007267	cell-cell signaling	2.19	0.015	-2.33	0.013
GO:0031589	cell-substrate adhesion	-1.76	0.015	2.14	0.009
GO:0001775	cell activation	1.5	0.015	2.51	0.009
GO:0002263	cell activation involved in immune response	1.95	0.015	2.46	0.009
GO:0033627	cell adhesion mediated by integrin	1.04	0.568	2.23	0.009
GO:0060326	cell chemotaxis	2.24	0.015	2.65	0.009
GO:0021533	cell differentiation in hindbrain	1.25	0.342	-2.02	0.01
GO:0001906	cell killing	2.02	0.015	2.33	0.009
GO:0016477	cell migration	1.43	0.015	2	0.009
GO:0048667	cell morphogenesis involved in neuron differentiation	1.92	0.015	-2.34	0.012
GO:0032990	cell part morphogenesis	1.69	0.015	-2.21	0.013
GO:0048858	cell projection morphogenesis	1.73	0.015	-2.27	0.013
GO:0008283	cell proliferation	-1.27	0.015	2.12	0.009
GO:1990748	cellular detoxification	-1.43	0.188	2	0.009
GO:0045123	cellular extravasation	2.2	0.015	2.23	0.009
GO:0071804	cellular potassium ion transport	1.81	0.015	-2.45	0.01
GO:0071216	cellular response to biotic stimulus	1.99	0.015	2.58	0.009
GO:0071345	cellular response to cytokine stimulus	2.04	0.015	2.51	0.009
GO:0035690	cellular response to drug	2.19	0.015	1.25	0.239
GO:0070301	cellular response to hydrogen peroxide	1.56	0.053	2.12	0.009
GO:0071346	cellular response to interferon-gamma	2.33	0.015	2.18	0.009
GO:0071347	cellular response to interleukin-1	2.38	0.015	2.35	0.009
GO:0071396	cellular response to lipid	1.39	0.015	2.08	0.009
GO:0071222	cellular response to lipopolysaccharide	2.06	0.015	2.49	0.009
GO:0071219	cellular response to molecule of bacterial origin	2.05	0.015	2.53	0.009
GO:0034614	cellular response to reactive oxygen species	1.45	0.066	2.21	0.009
GO:0071356	cellular response to tumor necrosis factor	2.04	0.015	2.27	0.009
GO:0071466	cellular response to xenobiotic stimulus	-2.02	0.015	0.97	0.63
GO:0021697	cerebellar cortex formation	1.55	0.109	-2.04	0.01
GO:0021549	cerebellum development	1.32	0.127	-2.02	0.01

GO:0070098	chemokine-mediated signaling pathway	2.43	0.015	2.15	0.009
GO:0050755	chemokine metabolic process	1.98	0.015	2.02	0.009
GO:0032602	chemokine production	1.43	0.103	2.26	0.009
GO:0006935	chemotaxis	2.09	0.015	1.94	0.009
GO:0002544	chronic inflammatory response	2.27	0.015	1.99	0.009
GO:0072359	circulatory system development	-1.68	0.015	2.04	0.009
GO:0050817	coagulation	-1.22	0.224	2.21	0.009
GO:0050890	cognition	2.29	0.015	-2.24	0.01
GO:0030574	collagen catabolic process	-1.02	0.598	2.05	0.009
GO:0030199	collagen fibril organization	-2.23	0.015	2.09	0.009
GO:0032963	collagen metabolic process	-1.16	0.382	2.23	0.009
GO:0006956	complement activation	1.16	0.419	2.05	0.009
GO:0060026	convergent extension	-2.01	0.015	0.6	0.975
GO:0019221	cytokine-mediated signaling pathway	2.18	0.015	2.38	0.009
GO:0042089	cytokine biosynthetic process	1.85	0.024	2.58	0.009
GO:0042107	cytokine metabolic process	1.99	0.015	2.59	0.009
GO:0001816	cytokine production	1.6	0.015	2.62	0.009
GO:0002367	cytokine production involved in immune response	1.36	0.158	2.1	0.009
GO:0050663	cytokine secretion	1.74	0.015	2.31	0.009
GO:0006952	defense response	1.82	0.015	2.53	0.009
GO:0042742	defense response to bacterium	1.78	0.015	2.6	0.009
GO:0050830	defense response to Gram-positive bacterium	1.51	0.058	2.32	0.009
GO:0098542	defense response to other organism	1.45	0.015	2.36	0.009
GO:0042832	defense response to protozoan	2.24	0.015	2	0.009
GO:0016358	dendrite development	2	0.015	-2.16	0.01
GO:0097484	dendrite extension	2.11	0.015	-2.23	0.01
GO:0048813	dendrite morphogenesis	2.07	0.015	-2.35	0.01
GO:0097028	dendritic cell differentiation	1.24	0.348	2.03	0.009
GO:0060996	dendritic spine development	1.82	0.024	-2.24	0.01
GO:0060997	dendritic spine morphogenesis	2.02	0.015	-2.38	0.01
GO:0097061	dendritic spine organization	1.9	0.015	-2.28	0.01
GO:0009595	detection of biotic stimulus	0.9	0.736	2.15	0.009
GO:0098581	detection of external biotic stimulus	0.96	0.672	2.16	0.009
GO:0098543	detection of other organism	1.34	0.295	2.02	0.009
GO:0098754	detoxification	-1.09	0.528	2.24	0.009
GO:0055123	digestive system development	-2.15	0.015	1.44	0.033
GO:0048565	digestive tract development	-2.09	0.015	1.47	0.026
GO:0051852	disruption by host of symbiont cells	2.01	0.015	1.88	0.009
GO:0044364	disruption of cells of other organism	2.15	0.015	1.89	0.013
GO:0051818	disruption of cells of other organism involved in symbiotic interaction	2.05	0.015	1.87	0.018
GO:0006261	DNA-dependent DNA replication	-0.93	0.744	2.21	0.009
GO:0006260	DNA replication	-1.08	0.436	2.3	0.009
GO:0006270	DNA replication initiation	0.7	0.951	2.23	0.009
GO:0014046	dopamine secretion	1.33	0.236	-2.11	0.01
GO:0015872	dopamine transport	1.29	0.261	-2.04	0.01
GO:0017144	drug metabolic process	-2.06	0.015	0.95	0.654
GO:0035113	embryonic appendage morphogenesis	-2.03	0.015	1.2	0.219
GO:0030326	embryonic limb morphogenesis	-2.03	0.015	1.2	0.219
GO:0035987	endodermal cell differentiation	-1.25	0.317	2.06	0.009
GO:0043542	endothelial cell migration	-1.47	0.043	2.01	0.009
GO:0051806	entry into cell of other organism involved in symbiotic interaction	-1.17	0.381	2.12	0.009
GO:0044409	entry into host	-1.17	0.381	2.12	0.009
GO:0030260	entry into host cell	-1.17	0.381	2.12	0.009
GO:0051828	entry into other organism involved in symbiotic interaction	-1.17	0.381	2.12	0.009
GO:0048245	eosinophil chemotaxis	2.25	0.015	1.71	0.032
GO:0072677	eosinophil migration	2.36	0.015	1.77	0.018
GO:0001837	epithelial to mesenchymal transition	-2.06	0.015	1.57	0.022
GO:0097480	establishment of synaptic vesicle localization	2.04	0.015	-2.65	0.01
GO:0051650	establishment of vesicle localization	1.92	0.015	-2.4	0.01
GO:0060079	excitatory postsynaptic potential	2.22	0.015	-2.63	0.01
GO:0006887	exocytosis	2.35	0.015	-1.87	0.011
GO:0035640	exploration behavior	1.61	0.089	-2.26	0.01
GO:0085029	extracellular matrix assembly	-2.08	0.015	1.44	0.116
GO:0030198	extracellular matrix organization	-2.09	0.015	2.18	0.009

GO:0043062	extracellular structure organization	-2.09	0.015	2.17	0.009
GO:0006635	fatty acid beta-oxidation	-2.17	0.015		
GO:0009062	fatty acid catabolic process	-2.07	0.015		
GO:1901570	fatty acid derivative biosynthetic process	-0.95	0.71	2.13	0.009
GO:1901568	fatty acid derivative metabolic process	-1.02	0.597	2.1	0.009
GO:0019395	fatty acid oxidation	-2.17	0.015	0.88	0.777
GO:0042596	fear response	1.54	0.064	-2.26	0.01
GO:0001660	fever generation	2.17	0.024	1.8	0.033
GO:0042730	fibrinolysis	1.52	0.161	2.12	0.009
GO:0048144	fibroblast proliferation	-1.28	0.206	2.15	0.009
GO:0007216	G-protein coupled glutamate receptor signaling pathway	2.05	0.015	-2.08	0.01
GO:0007214	gamma-aminobutyric acid signaling pathway	1.89	0.015	-2.26	0.01
GO:0007215	glutamate receptor signaling pathway	2.56	0.015	-2.83	0.01
GO:0014047	glutamate secretion	2.09	0.015	-1.54	0.057
GO:0036230	granulocyte activation	1.8	0.039	2.06	0.013
GO:0071621	granulocyte chemotaxis	2.59	0.015	2.65	0.009
GO:0097530	granulocyte migration	2.6	0.015	2.68	0.009
GO:0031649	heat generation	2.1	0.015	1.69	0.046
GO:0048534	hematopoietic or lymphoid organ development	1.13	0.208	2.08	0.009
GO:0030097	hemopoiesis	1.12	0.217	2.09	0.009
GO:0007599	hemostasis	-1.25	0.192	2.23	0.009
GO:0034113	heterotypic cell-cell adhesion	1.57	0.094	2.26	0.009
GO:0021766	hippocampus development	1.61	0.032	-2.18	0.01
GO:0007156	homophilic cell adhesion via plasma membrane adhesion molecules	1.44	0.073	-2.11	0.01
GO:0034109	homotypic cell-cell adhesion	1.52	0.015	2.42	0.009
GO:0006959	humoral immune response	1.31	0.148	2.48	0.009
GO:0002455	humoral immune response mediated by circulating immunoglobulin	1.44	0.163	2.12	0.009
GO:0002524	hypersensitivity	1.77	0.024	2.1	0.009
GO:0007249	I-kappaB kinase/NF-kappaB signaling	1.18	0.242	2.32	0.009
GO:0046456	icosanoid biosynthetic process	-0.95	0.71	2.13	0.009
GO:0006690	icosanoid metabolic process	-1.02	0.597	2.1	0.009
GO:0002252	immune effector process	1.47	0.016	2.52	0.009
GO:0006955	immune response	1.81	0.015	2.73	0.009
GO:0002429	immune response-activating cell surface receptor signaling pathway	1.55	0.015	2.15	0.009
GO:0002757	immune response-activating signal transduction	1.38	0.033	2.45	0.009
GO:0002768	immune response-regulating cell surface receptor signaling pathway	1.63	0.015	2.34	0.009
GO:0002764	immune response-regulating signaling pathway	1.44	0.024	2.55	0.009
GO:0002520	immune system development	1.15	0.111	2.08	0.009
GO:0002376	immune system process	1.45	0.015	2.47	0.009
GO:0016064	immunoglobulin mediated immune response	1.18	0.324	2.53	0.009
GO:0002377	immunoglobulin production	1.12	0.411	2.19	0.009
GO:0002381	immunoglobulin production involved in immunoglobulin mediated immune response	0.82	0.885	2.01	0.009
GO:0006954	inflammatory response	2.03	0.015	2.68	0.009
GO:0002437	inflammatory response to antigenic stimulus	2.18	0.015	2.13	0.009
GO:0045087	innate immune response	1.88	0.015	2.75	0.009
GO:0002758	innate immune response-activating signal transduction	1.39	0.126	2.47	0.009
GO:0098662	inorganic cation transmembrane transport	1.64	0.015	-2.23	0.013
GO:0098660	inorganic ion transmembrane transport	1.6	0.015	-2.27	0.013
GO:0007229	integrin-mediated signaling pathway	-1.18	0.32	2.19	0.009
GO:0051701	interaction with host	1.21	0.284	2.22	0.009
GO:0051702	interaction with symbiont	2.07	0.015	2.07	0.009
GO:0032608	interferon-beta production	-1.03	0.597	2.13	0.009
GO:0042095	interferon-gamma biosynthetic process	-0.77	0.875	2.01	0.009
GO:0032609	interferon-gamma production	1.79	0.015	2.21	0.009
GO:0032611	interleukin-1 beta production	1.85	0.024	2.26	0.009
GO:0050702	interleukin-1 beta secretion	1.42	0.202	2.09	0.009
GO:0032612	interleukin-1 production	1.68	0.038	2.24	0.009
GO:0050701	interleukin-1 secretion	1.2	0.392	2	0.009
GO:0032613	interleukin-10 production	1.69	0.031	2.14	0.009
GO:0032615	interleukin-12 production	1.45	0.152	2.22	0.009
GO:0042226	interleukin-6 biosynthetic process	1.35	0.284	2	0.009

GO:0032635	interleukin-6 production	1.33	0.157	2.24	0.009
GO:0032637	interleukin-8 production	1.62	0.046	2.52	0.009
GO:0072606	interleukin-8 secretion	1.21	0.41	2.07	0.009
GO:0044419	interspecies interaction between organisms	1.43	0.015	2.34	0.009
GO:0008630	intrinsic apoptotic signaling pathway in response to DNA damage	-1.24	0.224	2.13	0.009
GO:0034220	ion transmembrane transport	1.6	0.015	-2.09	0.013
GO:0035235	ionotropic glutamate receptor signaling pathway	2.07	0.024	-2.19	0.01
GO:0051873	killing by host of symbiont cells	2.01	0.015	1.88	0.009
GO:0051883	killing of cells in other organism involved in symbiotic interaction	2.05	0.015	1.87	0.018
GO:0031640	killing of cells of other organism	2.15	0.015	1.89	0.013
GO:0007612	learning	2.3	0.015	-2.41	0.01
GO:0007611	learning or memory	2.25	0.015	-2.35	0.01
GO:0045321	leukocyte activation	1.49	0.015	2.53	0.009
GO:0002366	leukocyte activation involved in immune response	1.95	0.015	2.46	0.009
GO:0070486	leukocyte aggregation	1.59	0.015	2.51	0.009
GO:0071887	leukocyte apoptotic process	1.18	0.319	2.46	0.009
GO:0007159	leukocyte cell-cell adhesion	1.69	0.015	2.53	0.009
GO:0030595	leukocyte chemotaxis	2.48	0.015	2.72	0.009
GO:0043299	leukocyte degranulation	1.82	0.015	2.07	0.009
GO:0002521	leukocyte differentiation	1.21	0.09	2.29	0.009
GO:0001776	leukocyte homeostasis	1.44	0.102	2.15	0.009
GO:0001909	leukocyte mediated cytotoxicity	1.98	0.015	2.37	0.009
GO:0002443	leukocyte mediated immunity	1.78	0.016	2.63	0.009
GO:0050900	leukocyte migration	2.37	0.015	2.74	0.009
GO:0070661	leukocyte proliferation	1.52	0.015	2.53	0.009
GO:0021761	limbic system development	1.67	0.015	-2.09	0.01
GO:0034440	lipid oxidation	-2.15	0.015	0.92	0.731
GO:0031663	lipopolysaccharide-mediated signaling pathway	2.02	0.015	2.15	0.009
GO:0007626	locomotory behavior	1.9	0.015	-2.66	0.011
GO:0007616	long-term memory	1.76	0.024	-2.4	0.01
GO:0060291	long-term synaptic potentiation	1.97	0.015	-2.33	0.01
GO:0060292	long term synaptic depression	2.09	0.015	-1.85	0.01
GO:0046649	lymphocyte activation	1.41	0.015	2.45	0.009
GO:0002285	lymphocyte activation involved in immune response	1.84	0.015	2.21	0.009
GO:0071593	lymphocyte aggregation	1.45	0.015	2.48	0.009
GO:0070227	lymphocyte apoptotic process	-0.99	0.646	2.37	0.009
GO:0048247	lymphocyte chemotaxis	2.45	0.015	2.13	0.009
GO:0030098	lymphocyte differentiation	1.16	0.233	2.2	0.009
GO:0002449	lymphocyte mediated immunity	1.41	0.051	2.45	0.009
GO:0072676	lymphocyte migration	2.34	0.015	2.37	0.009
GO:0046651	lymphocyte proliferation	1.45	0.015	2.54	0.009
GO:0042116	macrophage activation	-1.19	0.375	2.16	0.009
GO:0048246	macrophage chemotaxis	1.77	0.04	2.25	0.009
GO:0030225	macrophage differentiation	0.93	0.737	2.18	0.009
GO:0045576	mast cell activation	1.54	0.054	2.01	0.009
GO:0051899	membrane depolarization	1.83	0.015	-2.33	0.01
GO:0086010	membrane depolarization during action potential	2	0.015	-2.37	0.01
GO:0010324	membrane invagination	1.1	0.492	2.36	0.009
GO:0007613	memory	2.02	0.015	-2.2	0.01
GO:0014031	mesenchymal cell development	-2.11	0.015	1.59	0.009
GO:0048762	mesenchymal cell differentiation	-2.1	0.015	1.59	0.009
GO:0072132	mesenchyme morphogenesis	-2.1	0.015	1.76	0.013
GO:0022037	metencephalon development	1.34	0.121	-2.06	0.01
GO:0007067	mitotic nuclear division	-1.17	0.23	2.03	0.009
GO:0051851	modification by host of symbiont morphology or physiology	2.07	0.015	2.06	0.009
GO:0035821	modification of morphology or physiology of other organism	2.06	0.015	2.23	0.009
GO:0051817	modification of morphology or physiology of other organism involved in symbiotic interaction	2.01	0.015	2.23	0.009
GO:0072337	modified amino acid transport	-2.03	0.015	-1.57	0.053
GO:0098815	modulation of excitatory postsynaptic potential	1.88	0.031	-2.49	0.01
GO:0050804	modulation of synaptic transmission	2.47	0.015	-2.67	0.011
GO:0072329	monocarboxylic acid catabolic process	-2.09	0.015		
GO:0002548	monocyte chemotaxis	2.44	0.015	2.28	0.009
GO:0032943	mononuclear cell proliferation	1.44	0.024	2.55	0.009

GO:0052192	movement in environment of other organism involved in symbiotic interaction	-1.17	0.381	2.12	0.009
GO:0052126	movement in host environment	-1.17	0.381	2.12	0.009
GO:0044764	multi-organism cellular process	-1.18	0.251	2.27	0.009
GO:0044243	multicellular organismal catabolic process	-1.05	0.575	2.05	0.009
GO:0044259	multicellular organismal macromolecule metabolic process	-1.14	0.429	2.21	0.009
GO:0044236	multicellular organismal metabolic process	-1.17	0.387	2.21	0.009
GO:0035637	multicellular organismal signaling	1.72	0.024	-2.12	0.01
GO:0002275	myeloid cell activation involved in immune response	1.81	0.015	2.14	0.009
GO:0001773	myeloid dendritic cell activation	1.14	0.475	2.05	0.009
GO:0002274	myeloid leukocyte activation	1.66	0.015	2.56	0.009
GO:0002573	myeloid leukocyte differentiation	1.15	0.336	2.18	0.009
GO:0002444	myeloid leukocyte mediated immunity	1.93	0.015	2.32	0.009
GO:0097529	myeloid leukocyte migration	2.46	0.015	2.7	0.009
GO:0070266	necroptotic process	1.18	0.41	2.01	0.009
GO:0070265	necrotic cell death	0.96	0.697	2.02	0.009
GO:0016525	negative regulation of angiogenesis	1.45	0.095	2.07	0.009
GO:2000181	negative regulation of blood vessel morphogenesis	1.44	0.102	2.15	0.009
GO:0030514	negative regulation of BMP signaling pathway	-2.02	0.015	0.82	0.865
GO:0090090	negative regulation of canonical Wnt signaling pathway	-2.13	0.015	1.17	0.319
GO:0022408	negative regulation of cell-cell adhesion	-1.01	0.6	2.06	0.009
GO:0050866	negative regulation of cell activation	1.19	0.305	2.18	0.009
GO:0007162	negative regulation of cell adhesion	-1.16	0.287	2.08	0.009
GO:0042036	negative regulation of cytokine biosynthetic process	1.66	0.052	2.06	0.009
GO:0001818	negative regulation of cytokine production	1.36	0.077	2.38	0.009
GO:0031348	negative regulation of defense response	1.2	0.269	2.17	0.009
GO:0010951	negative regulation of endopeptidase activity	1.61	0.024	2.17	0.009
GO:1902042	negative regulation of extrinsic apoptotic signaling pathway via death domain receptors	1.81	0.031	2.05	0.009
GO:0034111	negative regulation of homotypic cell-cell adhesion	0.9	0.787	2.03	0.009
GO:0002698	negative regulation of immune effector process	1.08	0.494	2.19	0.009
GO:0050777	negative regulation of immune response	1.38	0.114	2.36	0.009
GO:0002683	negative regulation of immune system process	1.2	0.179	2.35	0.009
GO:0050728	negative regulation of inflammatory response	1.36	0.141	2.11	0.009
GO:0002695	negative regulation of leukocyte activation	1.29	0.169	2.28	0.009
GO:2000107	negative regulation of leukocyte apoptotic process	-1.08	0.528	2.39	0.009
GO:1903038	negative regulation of leukocyte cell-cell adhesion	0.89	0.81	2.14	0.009
GO:0002704	negative regulation of leukocyte mediated immunity	1.57	0.085	2.07	0.009
GO:0070664	negative regulation of leukocyte proliferation	1.27	0.251	2.13	0.009
GO:0051250	negative regulation of lymphocyte activation	1.26	0.232	2.19	0.009
GO:0070229	negative regulation of lymphocyte apoptotic process	-0.9	0.745	2.1	0.009
GO:0050672	negative regulation of lymphocyte proliferation	1.11	0.458	2.16	0.009
GO:0032945	negative regulation of mononuclear cell proliferation	1.11	0.458	2.16	0.009
GO:0043901	negative regulation of multi-organism process	1.42	0.083	2.36	0.009
GO:0032891	negative regulation of organic acid transport	2.01	0.015	1.2	0.357
GO:0010466	negative regulation of peptidase activity	1.52	0.024	2.14	0.009
GO:0002701	negative regulation of production of molecular mediator of immune response	-0.91	0.74	2.01	0.009
GO:1903318	negative regulation of protein maturation	1.52	0.083	2.06	0.009
GO:0010955	negative regulation of protein processing	1.52	0.083	2.06	0.009
GO:1903035	negative regulation of response to wounding	1.35	0.12	2.18	0.009
GO:0050805	negative regulation of synaptic transmission	2.02	0.015	-2.14	0.01
GO:0050868	negative regulation of T cell activation	1.01	0.615	2.16	0.009
GO:1901343	negative regulation of vasculature development	1.37	0.149	2.19	0.009
GO:0045071	negative regulation of viral genome replication	-1.02	0.605	2.02	0.009
GO:1903901	negative regulation of viral life cycle	1.55	0.075	2.29	0.009
GO:0048525	negative regulation of viral process	1.47	0.096	2.31	0.009
GO:0030178	negative regulation of Wnt signaling pathway	-2.16	0.015	1.17	0.274
GO:0003407	neural retina development	1.29	0.24	-2.09	0.01
GO:0050905	neuromuscular process	2.02	0.015	-2.34	0.01
GO:0050885	neuromuscular process controlling balance	1.33	0.218	-2.05	0.01
GO:0007270	neuron-neuron synaptic transmission	2.31	0.015	-2.99	0.01
GO:0048666	neuron development	1.83	0.015	-2.07	0.013
GO:0031175	neuron projection development	1.86	0.015	-2.07	0.013
GO:0097485	neuron projection guidance	1.48	0.024	-2.11	0.011
GO:0048812	neuron projection morphogenesis	2.01	0.015	-2.39	0.012

GO:0008038	neuron recognition	1.89	0.015	-2.19	0.01
GO:0019228	neuronal action potential	1.96	0.015	-2.08	0.01
GO:0007269	neurotransmitter secretion	2.39	0.015	-2.93	0.01
GO:0006836	neurotransmitter transport	2.15	0.015	-2.83	0.011
GO:0030593	neutrophil chemotaxis	2.58	0.015	2.63	0.009
GO:0002446	neutrophil mediated immunity	1.93	0.015	2.21	0.009
GO:1990266	neutrophil migration	2.6	0.015	2.67	0.009
GO:0006809	nitric oxide biosynthetic process	1.85	0.024	2.13	0.009
GO:0046209	nitric oxide metabolic process	1.8	0.024	2.13	0.009
GO:0035567	non-canonical Wnt signaling pathway	-2.16	0.015	1.37	0.164
GO:0002251	organ or tissue specific immune response	-0.94	0.712	2.04	0.009
GO:0031100	organ regeneration	-1.14	0.357	2.21	0.009
GO:0007567	parturition	1.63	0.087	2.03	0.009
GO:0002221	pattern recognition receptor signaling pathway	1.4	0.111	2.45	0.009
GO:0006909	phagocytosis	1.76	0.015	2.6	0.009
GO:0006911	phagocytosis, engulfment	1.2	0.405	2.42	0.009
GO:0006910	phagocytosis, recognition	1.09	0.536	2.06	0.009
GO:0042104	positive regulation of activated T cell proliferation	-1.27	0.347	2.01	0.009
GO:0002675	positive regulation of acute inflammatory response	1.94	0.015	2.16	0.009
GO:0002821	positive regulation of adaptive immune response	1.51	0.063	2.4	0.009
GO:0002824	positive regulation of adaptive immune response based on somatic recombination of immune receptors built from immunoglobulin superfamily domains	1.53	0.049	2.36	0.009
GO:0046635	positive regulation of alpha-beta T cell activation	1.43	0.128	2.2	0.009
GO:0051954	positive regulation of amine transport	1.58	0.058	-2.03	0.01
GO:0045766	positive regulation of angiogenesis	1.43	0.08	2.48	0.009
GO:0050871	positive regulation of B cell activation	1.17	0.388	2.14	0.009
GO:0002714	positive regulation of B cell mediated immunity	1.47	0.091	2.24	0.009
GO:0048520	positive regulation of behavior	2.17	0.015	2.26	0.009
GO:0030194	positive regulation of blood coagulation	2.07	0.015	2.16	0.009
GO:0045956	positive regulation of calcium ion-dependent exocytosis	1.88	0.015	-2.13	0.01
GO:0022409	positive regulation of cell-cell adhesion	1.7	0.015	2.38	0.009
GO:0050867	positive regulation of cell activation	1.67	0.015	2.49	0.009
GO:0045785	positive regulation of cell adhesion	1.42	0.024	2.4	0.009
GO:0031343	positive regulation of cell killing	2.11	0.015	2.1	0.009
GO:0030335	positive regulation of cell migration	1.77	0.015	2.33	0.009
GO:2000147	positive regulation of cell motility	1.76	0.015	2.31	0.009
GO:0008284	positive regulation of cell proliferation	-1.16	0.124	2.09	0.009
GO:0051272	positive regulation of cellular component movement	1.75	0.015	2.29	0.009
GO:0032722	positive regulation of chemokine production	1.47	0.1	2.11	0.009
GO:0050921	positive regulation of chemotaxis	2.21	0.015	2.44	0.009
GO:0050820	positive regulation of coagulation	1.99	0.015	2.23	0.009
GO:0042108	positive regulation of cytokine biosynthetic process	1.84	0.015	2.46	0.009
GO:0001819	positive regulation of cytokine production	1.79	0.015	2.58	0.009
GO:0050715	positive regulation of cytokine secretion	1.79	0.015	2.03	0.009
GO:0031349	positive regulation of defense response	1.83	0.015	2.6	0.009
GO:1900006	positive regulation of dendrite development	1.64	0.024	-2.05	0.01
GO:1903861	positive regulation of dendrite extension	2.11	0.015	-2.3	0.01
GO:0060999	positive regulation of dendritic spine development	1.52	0.105	-2.25	0.01
GO:0061003	positive regulation of dendritic spine morphogenesis	1.7	0.08	-2.17	0.01
GO:0045807	positive regulation of endocytosis	1.96	0.015	2.03	0.009
GO:0070374	positive regulation of ERK1 and ERK2 cascade	2.18	0.015	1.89	0.009
GO:2000463	positive regulation of excitatory postsynaptic potential	2	0.015	-2.41	0.01
GO:0048146	positive regulation of fibroblast proliferation	-1.22	0.327	2.22	0.009
GO:0071624	positive regulation of granulocyte chemotaxis	1.7	0.052	2.18	0.009
GO:1903708	positive regulation of hemopoiesis	1.49	0.024	2.21	0.009
GO:1900048	positive regulation of hemostasis	2.07	0.015	2.16	0.009
GO:0034112	positive regulation of homotypic cell-cell adhesion	1.74	0.015	2.41	0.009
GO:0043123	positive regulation of I-kappaB kinase/NF-kappaB signaling	-1.06	0.52	2.38	0.009
GO:0002699	positive regulation of immune effector process	1.86	0.015	2.51	0.009
GO:0050778	positive regulation of immune response	1.29	0.051	2.59	0.009
GO:0002684	positive regulation of immune system process	1.75	0.015	2.69	0.009
GO:0002891	positive regulation of immunoglobulin mediated immune response	1.47	0.091	2.24	0.009
GO:0002639	positive regulation of immunoglobulin production	1.2	0.389	2.1	0.009
GO:0050729	positive regulation of inflammatory response	2.16	0.015	2.41	0.009

GO:0045089	positive regulation of innate immune response	1.17	0.291	2.48	0.009
GO:0032729	positive regulation of interferon-gamma production	1.67	0.024	2.07	0.009
GO:0032731	positive regulation of interleukin-1 beta production	1.31	0.289	2.16	0.009
GO:0032732	positive regulation of interleukin-1 production	1.25	0.328	2.25	0.009
GO:0032755	positive regulation of interleukin-6 production	1.48	0.08	2.28	0.009
GO:0032757	positive regulation of interleukin-8 production	1.65	0.053	2.47	0.009
GO:0002696	positive regulation of leukocyte activation	1.53	0.015	2.48	0.009
GO:1903039	positive regulation of leukocyte cell-cell adhesion	1.69	0.015	2.52	0.009
GO:0002690	positive regulation of leukocyte chemotaxis	2.33	0.015	2.59	0.009
GO:1902107	positive regulation of leukocyte differentiation	1.51	0.024	2.25	0.009
GO:0001912	positive regulation of leukocyte mediated cytotoxicity	2.05	0.015	2.1	0.009
GO:0002705	positive regulation of leukocyte mediated immunity	1.97	0.015	2.26	0.009
GO:0002687	positive regulation of leukocyte migration	2.4	0.015	2.66	0.009
GO:0070665	positive regulation of leukocyte proliferation	1.46	0.047	2.42	0.009
GO:0040017	positive regulation of locomotion	1.75	0.015	2.28	0.009
GO:0051251	positive regulation of lymphocyte activation	1.48	0.024	2.41	0.009
GO:0045621	positive regulation of lymphocyte differentiation	1.47	0.094	2.08	0.009
GO:0002708	positive regulation of lymphocyte mediated immunity	1.53	0.033	2.09	0.009
GO:2000403	positive regulation of lymphocyte migration	2	0.015	2.08	0.009
GO:0050671	positive regulation of lymphocyte proliferation	1.5	0.04	2.4	0.009
GO:0045651	positive regulation of macrophage differentiation	0.89	0.746	2	0.009
GO:0090026	positive regulation of monocyte chemotaxis	2.13	0.015	2.21	0.009
GO:0032946	positive regulation of mononuclear cell proliferation	1.49	0.039	2.41	0.009
GO:0043902	positive regulation of multi-organism process	0.81	0.953	2.07	0.009
GO:0045639	positive regulation of myeloid cell differentiation	1.05	0.552	2.01	0.009
GO:0002763	positive regulation of myeloid leukocyte differentiation	1.21	0.353	2.08	0.009
GO:0002888	positive regulation of myeloid leukocyte mediated immunity	1.79	0.039	2.09	0.009
GO:0010976	positive regulation of neuron projection development	1.73	0.015	-2.16	0.011
GO:0001956	positive regulation of neurotransmitter secretion	1.69	0.065	-2.16	0.01
GO:0051590	positive regulation of neurotransmitter transport	1.68	0.047	-2.16	0.01
GO:0090023	positive regulation of neutrophil chemotaxis	1.69	0.031	2.17	0.009
GO:1902624	positive regulation of neutrophil migration	1.82	0.031	2.24	0.009
GO:0051092	positive regulation of NF-kappaB transcription factor activity	1.18	0.322	2.03	0.009
GO:0045429	positive regulation of nitric oxide biosynthetic process	2.12	0.015	2	0.009
GO:1904407	positive regulation of nitric oxide metabolic process	2.12	0.015	2	0.009
GO:0050766	positive regulation of phagocytosis	1.97	0.015	2.45	0.009
GO:0002702	positive regulation of production of molecular mediator of immune response	1.29	0.227	2.21	0.009
GO:1903428	positive regulation of reactive oxygen species biosynthetic process	2.09	0.024	2.01	0.009
GO:2000379	positive regulation of reactive oxygen species metabolic process	2.07	0.015	2.32	0.009
GO:0002833	positive regulation of response to biotic stimulus	1.38	0.2	2.06	0.009
GO:0032103	positive regulation of response to external stimulus	2.11	0.015	2.53	0.009
GO:0048584	positive regulation of response to stimulus	1.37	0.015	2.04	0.009
GO:1903036	positive regulation of response to wounding	2.19	0.015	2.4	0.009
GO:0051965	positive regulation of synapse assembly	2.07	0.015	-2.61	0.01
GO:0050806	positive regulation of synaptic transmission	2.36	0.015	-2.57	0.01
GO:0051968	positive regulation of synaptic transmission, glutamatergic	1.9	0.024	-2.09	0.01
GO:0002827	positive regulation of T-helper 1 type immune response	1.68	0.083	2.04	0.009
GO:0050870	positive regulation of T cell activation	1.63	0.015	2.48	0.009
GO:0045582	positive regulation of T cell differentiation	1.68	0.041	2.02	0.009
GO:2000406	positive regulation of T cell migration	1.55	0.134	2	0.009
GO:0042102	positive regulation of T cell proliferation	1.6	0.024	2.46	0.009
GO:0042535	positive regulation of tumor necrosis factor biosynthetic process	1.64	0.07	2.19	0.009
GO:0032760	positive regulation of tumor necrosis factor production	1.81	0.015	2.7	0.009
GO:1903557	positive regulation of tumor necrosis factor superfamily cytokine production	1.78	0.015	2.72	0.009
GO:0032481	positive regulation of type I interferon production	-1.19	0.385	2.05	0.009
GO:0010575	positive regulation of vascular endothelial growth factor production	1.88	0.015	2.14	0.009
GO:1904018	positive regulation of vasculature development	1.39	0.101	2.48	0.009
GO:0090303	positive regulation of wound healing	1.93	0.015	2.11	0.009
GO:0043368	positive T cell selection	1.48	0.14	2.06	0.009
GO:0071805	potassium ion transmembrane transport	1.81	0.015	-2.45	0.01

GO:0006813	potassium ion transport	1.88	0.015	-2.36	0.01
GO:0097090	presynaptic membrane organization	1.37	0.221	-2.2	0.01
GO:0002532	production of molecular mediator involved in inflammatory response	0.93	0.737	2.2	0.009
GO:0002440	production of molecular mediator of immune response	1.32	0.11	2.35	0.009
GO:0001516	prostaglandin biosynthetic process	0.85	0.814	2.01	0.009
GO:0006693	prostaglandin metabolic process	-0.93	0.72	2.21	0.009
GO:0046457	prostanoid biosynthetic process	0.85	0.814	2.01	0.009
GO:0006692	prostanoid metabolic process	-0.93	0.72	2.21	0.009
GO:0072376	protein activation cascade	1.1	0.474	2.16	0.009
GO:0035418	protein localization to synapse	1.78	0.059	-2.25	0.01
GO:2001057	reactive nitrogen species metabolic process	1.72	0.024	2.17	0.009
GO:1903409	reactive oxygen species biosynthetic process	1.76	0.024	2.12	0.009
GO:0072593	reactive oxygen species metabolic process	1.56	0.015	2.35	0.009
GO:0031099	regeneration	-1.25	0.166	2.1	0.009
GO:0002673	regulation of acute inflammatory response	1.66	0.015	2.16	0.009
GO:0002819	regulation of adaptive immune response	1.55	0.033	2.44	0.009
GO:0002822	regulation of adaptive immune response based on somatic recombination of immune receptors built from immunoglobulin superfamily domains	1.54	0.033	2.38	0.009
GO:2000311	regulation of alpha-amino-3-hydroxy-5-methyl-4-isoxazole propionate selective glutamate receptor activity	2.05	0.015	-2.16	0.01
GO:0046634	regulation of alpha-beta T cell activation	1.39	0.136	2.28	0.009
GO:0046640	regulation of alpha-beta T cell proliferation	1.42	0.167	2.05	0.009
GO:0051952	regulation of amine transport	1.93	0.015	-2.08	0.01
GO:0045765	regulation of angiogenesis	1.36	0.061	2.51	0.009
GO:0002577	regulation of antigen processing and presentation	1.18	0.466	2.16	0.009
GO:0050854	regulation of antigen receptor-mediated signaling pathway	-0.74	0.945	2.09	0.009
GO:0050864	regulation of B cell activation	1.35	0.123	2.22	0.009
GO:0002902	regulation of B cell apoptotic process	0.92	0.724	2.02	0.009
GO:0002712	regulation of B cell mediated immunity	1.51	0.07	2.16	0.009
GO:0030888	regulation of B cell proliferation	1.02	0.565	2.11	0.009
GO:0030193	regulation of blood coagulation	1.33	0.199	2.07	0.009
GO:0017158	regulation of calcium ion-dependent exocytosis	2.09	0.015	-2.34	0.01
GO:0050433	regulation of catecholamine secretion	1.53	0.082	-2.19	0.01
GO:0022407	regulation of cell-cell adhesion	1.43	0.015	2.29	0.009
GO:0010810	regulation of cell-substrate adhesion	-1.56	0.031	2.04	0.009
GO:0050865	regulation of cell activation	1.45	0.015	2.42	0.009
GO:0030155	regulation of cell adhesion	1.23	0.078	2.29	0.009
GO:0033628	regulation of cell adhesion mediated by integrin	1.04	0.589	2.33	0.009
GO:0031341	regulation of cell killing	1.95	0.015	2.07	0.009
GO:0030334	regulation of cell migration	1.47	0.015	2.19	0.009
GO:2000145	regulation of cell motility	1.43	0.015	2.19	0.009
GO:0042127	regulation of cell proliferation	-1.33	0.015	2.11	0.009
GO:0051270	regulation of cellular component movement	1.4	0.015	2.06	0.009
GO:0002691	regulation of cellular extravasation	1.93	0.015	2.03	0.009
GO:0032642	regulation of chemokine production	1.44	0.096	2.28	0.009
GO:0050920	regulation of chemotaxis	1.95	0.015	2.37	0.009
GO:0050818	regulation of coagulation	1.3	0.207	2.08	0.009
GO:2000116	regulation of cysteine-type endopeptidase activity	1.47	0.031	2	0.009
GO:0043281	regulation of cysteine-type endopeptidase activity involved in apoptotic process	1.44	0.046	2.04	0.009
GO:0001959	regulation of cytokine-mediated signaling pathway	-0.99	0.653	2.13	0.009
GO:0042035	regulation of cytokine biosynthetic process	1.91	0.024	2.57	0.009
GO:0001817	regulation of cytokine production	1.6	0.015	2.64	0.009
GO:0002718	regulation of cytokine production involved in immune response	1.07	0.504	2.13	0.009
GO:0050707	regulation of cytokine secretion	1.64	0.015	2.24	0.009
GO:0031347	regulation of defense response	1.57	0.016	2.38	0.009
GO:0050773	regulation of dendrite development	1.89	0.015	-2.23	0.01
GO:1903859	regulation of dendrite extension	2.11	0.015	-2.3	0.01
GO:0048814	regulation of dendrite morphogenesis	1.95	0.015	-2.28	0.01
GO:0060998	regulation of dendritic spine development	1.64	0.033	-2.2	0.01
GO:0061001	regulation of dendritic spine morphogenesis	1.81	0.024	-2.26	0.01
GO:0014059	regulation of dopamine secretion	1.33	0.236	-2.11	0.01
GO:0052548	regulation of endopeptidase activity	1.48	0.015	2.13	0.009

GO:0010717	regulation of epithelial to mesenchymal transition	-2.02	0.015	1.42	0.103
GO:0090175	regulation of establishment of planar polarity	-2.23	0.015	1.68	0.023
GO:0017157	regulation of exocytosis	2.22	0.015	-1.87	0.011
GO:1902041	regulation of extrinsic apoptotic signaling pathway via death domain receptors	1.64	0.024	2.03	0.009
GO:0048145	regulation of fibroblast proliferation	-1.24	0.25	2.14	0.009
GO:1900449	regulation of glutamate receptor signaling pathway	2.22	0.015	-2.5	0.01
GO:0014048	regulation of glutamate secretion	2	0.015	-1.35	0.19
GO:0071622	regulation of granulocyte chemotaxis	2.05	0.015	2.25	0.009
GO:1903706	regulation of hemopoiesis	1.23	0.129	2.07	0.009
GO:1900046	regulation of hemostasis	1.33	0.199	2.07	0.009
GO:0034110	regulation of homotypic cell-cell adhesion	1.48	0.015	2.34	0.009
GO:0002920	regulation of humoral immune response	1.08	0.512	2.11	0.009
GO:0043122	regulation of I-kappaB kinase/NF-kappaB signaling	1.04	0.512	2.3	0.009
GO:0002697	regulation of immune effector process	1.33	0.035	2.2	0.009
GO:0050776	regulation of immune response	1.28	0.043	2.66	0.009
GO:0002682	regulation of immune system process	1.5	0.016	2.45	0.009
GO:0002889	regulation of immunoglobulin mediated immune response	1.51	0.07	2.16	0.009
GO:0002637	regulation of immunoglobulin production	1.34	0.207	2.11	0.009
GO:0050727	regulation of inflammatory response	1.8	0.015	2.5	0.009
GO:0045088	regulation of innate immune response	1.22	0.236	2.53	0.009
GO:0032648	regulation of interferon-beta production	-1.04	0.58	2.1	0.009
GO:0032649	regulation of interferon-gamma production	1.72	0.015	2.25	0.009
GO:0032651	regulation of interleukin-1 beta production	1.46	0.138	2.15	0.009
GO:0032652	regulation of interleukin-1 production	1.33	0.216	2.19	0.009
GO:0032653	regulation of interleukin-10 production	1.65	0.039	2.09	0.009
GO:0032655	regulation of interleukin-12 production	1.52	0.091	2.22	0.009
GO:0045408	regulation of interleukin-6 biosynthetic process	1.35	0.269	2.01	0.009
GO:0032675	regulation of interleukin-6 production	1.33	0.158	2.25	0.009
GO:0032677	regulation of interleukin-8 production	1.65	0.053	2.52	0.009
GO:2000482	regulation of interleukin-8 secretion	1.23	0.405	2.03	0.009
GO:0034765	regulation of ion transmembrane transport	2.05	0.015	-2.26	0.012
GO:0032412	regulation of ion transmembrane transporter activity	1.71	0.015	-2.11	0.01
GO:0043269	regulation of ion transport	2.02	0.015	-1.95	0.012
GO:0002694	regulation of leukocyte activation	1.4	0.015	2.47	0.009
GO:2000106	regulation of leukocyte apoptotic process	-1.01	0.604	2.36	0.009
GO:1903037	regulation of leukocyte cell-cell adhesion	1.41	0.049	2.47	0.009
GO:0002688	regulation of leukocyte chemotaxis	2.22	0.015	2.61	0.009
GO:0043300	regulation of leukocyte degranulation	1.55	0.093	2.09	0.009
GO:1902105	regulation of leukocyte differentiation	1.24	0.178	2.21	0.009
GO:0001910	regulation of leukocyte mediated cytotoxicity	1.91	0.015	2.1	0.009
GO:0002703	regulation of leukocyte mediated immunity	1.84	0.015	2.49	0.009
GO:0002685	regulation of leukocyte migration	2.29	0.015	2.66	0.009
GO:0070663	regulation of leukocyte proliferation	1.52	0.015	2.55	0.009
GO:0040012	regulation of locomotion	1.5	0.015	2.14	0.009
GO:0048169	regulation of long-term neuronal synaptic plasticity	2.26	0.015	-2.21	0.01
GO:0051249	regulation of lymphocyte activation	1.39	0.025	2.44	0.009
GO:0070228	regulation of lymphocyte apoptotic process	-1.02	0.58	2.3	0.009
GO:1901623	regulation of lymphocyte chemotaxis	2.22	0.015	1.99	0.009
GO:0045619	regulation of lymphocyte differentiation	1.26	0.212	2.04	0.009
GO:0002706	regulation of lymphocyte mediated immunity	1.34	0.148	2.17	0.009
GO:2000401	regulation of lymphocyte migration	2.12	0.015	2.27	0.009
GO:0050670	regulation of lymphocyte proliferation	1.5	0.015	2.56	0.009
GO:0010758	regulation of macrophage chemotaxis	1.75	0.053	2.07	0.009
GO:0045649	regulation of macrophage differentiation	0.91	0.722	2.05	0.009
GO:0042391	regulation of membrane potential	2.08	0.015	-2.56	0.011
GO:0090025	regulation of monocyte chemotaxis	2.01	0.015	2.1	0.009
GO:0032944	regulation of mononuclear cell proliferation	1.49	0.015	2.57	0.009
GO:0043900	regulation of multi-organism process	1.14	0.277	2.07	0.009
GO:0002761	regulation of myeloid leukocyte differentiation	1.04	0.523	2.13	0.009
GO:0002886	regulation of myeloid leukocyte mediated immunity	1.8	0.024	2.3	0.009
GO:2000310	regulation of N-methyl-D-aspartate selective glutamate receptor activity	1.92	0.024	-2.33	0.01
GO:0010975	regulation of neuron projection development	1.82	0.015	-2.13	0.012
GO:0048168	regulation of neuronal synaptic plasticity	2.42	0.015	-2.22	0.01
GO:0001505	regulation of neurotransmitter levels	2.21	0.015	-2.78	0.01

GO:0046928	regulation of neurotransmitter secretion	2	0.015	-2.56	0.01
GO:0051588	regulation of neurotransmitter transport	1.94	0.015	-2.35	0.01
GO:0090022	regulation of neutrophil chemotaxis	1.67	0.038	2.11	0.009
GO:1902622	regulation of neutrophil migration	1.81	0.024	2.24	0.009
GO:0045428	regulation of nitric oxide biosynthetic process	1.9	0.024	2.11	0.009
GO:2000027	regulation of organ morphogenesis	-2.12	0.015	1.68	0.009
GO:0052547	regulation of peptidase activity	1.34	0.031	2.15	0.009
GO:0050764	regulation of phagocytosis	1.95	0.015	2.44	0.009
GO:0060078	regulation of postsynaptic membrane potential	2.29	0.015	-2.67	0.01
GO:0043266	regulation of potassium ion transport	1.44	0.091	-2.02	0.01
GO:0002700	regulation of production of molecular mediator of immune response	1.27	0.227	2.31	0.009
GO:1903317	regulation of protein maturation	1.22	0.307	2.06	0.009
GO:0070613	regulation of protein processing	1.23	0.292	2.07	0.009
GO:1903426	regulation of reactive oxygen species biosynthetic process	1.85	0.024	2.03	0.009
GO:2000377	regulation of reactive oxygen species metabolic process	1.78	0.015	2.23	0.009
GO:0060759	regulation of response to cytokine stimulus	-1	0.637	2.13	0.009
GO:0032101	regulation of response to external stimulus	1.6	0.016	2.09	0.009
GO:0080134	regulation of response to stress	1.37	0.015	2.02	0.009
GO:1903034	regulation of response to wounding	1.67	0.015	2.4	0.009
GO:0060390	regulation of SMAD protein import into nucleus	-2.08	0.015	1.81	0.013
GO:2000736	regulation of stem cell differentiation	-2.22	0.015	1.71	0.009
GO:2000036	regulation of stem cell population maintenance	-2.06	0.015	1.62	0.044
GO:0090322	regulation of superoxide metabolic process	1.22	0.396	2.14	0.009
GO:0043903	regulation of symbiosis, encompassing mutualism through parasitism	1.38	0.078	2.4	0.009
GO:0051963	regulation of synapse assembly	2.14	0.015	-2.72	0.01
GO:0050807	regulation of synapse organization	2	0.015	-2.61	0.01
GO:0050803	regulation of synapse structure or activity	2.42	0.015	-2.62	0.011
GO:0048167	regulation of synaptic plasticity	2.49	0.015	-2.45	0.01
GO:0032228	regulation of synaptic transmission, GABAergic	1.6	0.071	-2.12	0.01
GO:0051966	regulation of synaptic transmission, glutamatergic	1.95	0.015	-2.75	0.01
GO:2000300	regulation of synaptic vesicle exocytosis	1.94	0.031	-2.04	0.01
GO:1902803	regulation of synaptic vesicle transport	2	0.024	-2.16	0.01
GO:0002825	regulation of T-helper 1 type immune response	1.67	0.053	2.2	0.009
GO:0050863	regulation of T cell activation	1.39	0.056	2.46	0.009
GO:0070232	regulation of T cell apoptotic process	-1.36	0.2	2.03	0.009
GO:0045580	regulation of T cell differentiation	1.4	0.09	2.04	0.009
GO:2000404	regulation of T cell migration	1.5	0.144	2.1	0.009
GO:0042129	regulation of T cell proliferation	1.5	0.015	2.53	0.009
GO:0034121	regulation of toll-like receptor signaling pathway	1.26	0.319	2.04	0.009
GO:0034762	regulation of transmembrane transport	1.99	0.015	-2.24	0.011
GO:0022898	regulation of transmembrane transporter activity	1.67	0.015	-2.09	0.01
GO:0032409	regulation of transporter activity	1.62	0.015	-2.05	0.01
GO:0042534	regulation of tumor necrosis factor biosynthetic process	1.9	0.024	2.27	0.009
GO:0032680	regulation of tumor necrosis factor production	1.71	0.015	2.67	0.009
GO:1903555	regulation of tumor necrosis factor superfamily cytokine production	1.74	0.015	2.68	0.009
GO:0002828	regulation of type 2 immune response	1.81	0.024	2.09	0.009
GO:0032479	regulation of type I interferon production	1.01	0.616	2.23	0.009
GO:0010574	regulation of vascular endothelial growth factor production	2.29	0.015	2.24	0.009
GO:1901342	regulation of vasculature development	1.27	0.137	2.54	0.009
GO:0060627	regulation of vesicle-mediated transport	2.12	0.015	1.34	0.031
GO:0046596	regulation of viral entry into host cell	1.43	0.197	2.07	0.009
GO:0045069	regulation of viral genome replication	-0.94	0.722	2.11	0.009
GO:1903900	regulation of viral life cycle	1.27	0.185	2.28	0.009
GO:0050792	regulation of viral process	1.2	0.276	2.29	0.009
GO:0061041	regulation of wound healing	1.25	0.232	2.06	0.009
GO:0019430	removal of superoxide radicals	-1.08	0.559	2.11	0.009
GO:0033198	response to ATP	2.02	0.015	1.69	0.023
GO:0009617	response to bacterium	1.97	0.015	2.61	0.009
GO:0009607	response to biotic stimulus	1.72	0.015	2.48	0.009
GO:0042220	response to cocaine	1.75	0.024	-2.07	0.01
GO:0031960	response to corticosteroid	1.49	0.015	2.03	0.009
GO:0034097	response to cytokine	2.09	0.015	2.53	0.009
GO:0043207	response to external biotic stimulus	1.75	0.015	2.48	0.009

GO:0034405	response to fluid shear stress	1	0.612	2.02	0.009
GO:0060992	response to fungicide	1.61	0.065	-2.13	0.01
GO:0009620	response to fungus	2.27	0.015	2.24	0.009
GO:0010332	response to gamma radiation	2.15	0.015	2.14	0.009
GO:0051384	response to glucocorticoid	1.55	0.015	2.06	0.009
GO:0009629	response to gravity	2.05	0.015	1.35	0.22
GO:0042542	response to hydrogen peroxide	1.62	0.015	2.22	0.009
GO:0055093	response to hyperoxia	1.45	0.08	2.24	0.009
GO:0036296	response to increased oxygen levels	1.45	0.08	2.24	0.009
GO:0035455	response to interferon-alpha	-1.55	0.109	2.02	0.009
GO:0034341	response to interferon-gamma	2.18	0.015	2.38	0.009
GO:0070555	response to interleukin-1	2.36	0.015	2.22	0.009
GO:0070670	response to interleukin-4	1.35	0.255	2	0.009
GO:0070741	response to interleukin-6	1.86	0.024	2.01	0.009
GO:0010212	response to ionizing radiation	1.72	0.015	2.12	0.009
GO:0034616	response to laminar fluid shear stress	-0.98	0.652	2.01	0.009
GO:0033993	response to lipid	1.39	0.015	2.08	0.009
GO:0032496	response to lipopolysaccharide	2.01	0.015	2.54	0.009
GO:0055094	response to lipoprotein particle	1.97	0.015	2.02	0.009
GO:0002237	response to molecule of bacterial origin	1.98	0.015	2.56	0.009
GO:0051707	response to other organism	1.75	0.015	2.49	0.009
GO:0000305	response to oxygen radical	-1.17	0.463	2.03	0.009
GO:0036119	response to platelet-derived growth factor	1.56	0.115	2.01	0.009
GO:0001562	response to protozoan	2.2	0.015	1.97	0.009
GO:0000302	response to reactive oxygen species	1.6	0.024	2.23	0.009
GO:0006950	response to stress	1.26	0.015	2.09	0.009
GO:0000303	response to superoxide	-1.24	0.372	2.01	0.009
GO:0034612	response to tumor necrosis factor	2.04	0.015	2.34	0.009
GO:0009615	response to virus	1.13	0.317	2	0.009
GO:0009611	response to wounding	1.43	0.015	2.4	0.009
GO:0001878	response to yeast	2.06	0.015	1.81	0.009
GO:0010043	response to zinc ion	1.48	0.087	2.05	0.009
GO:0010842	retina layer formation	-1	0.61	-2.02	0.01
GO:0042573	retinoic acid metabolic process	-1.79	0.031	2.06	0.009
GO:0000028	ribosomal small subunit assembly	-1.05	0.563	2.04	0.009
GO:0042274	ribosomal small subunit biogenesis	0.95	0.709	2.12	0.009
GO:0042254	ribosome biogenesis	1.56	0.024	2.11	0.009
GO:0019233	sensory perception of pain	2.04	0.015	-1.79	0.01
GO:0007210	serotonin receptor signaling pathway	1.9	0.024	-2.04	0.01
GO:0023061	signal release	2.04	0.015	-2.23	0.012
GO:0044708	single-organism behavior	2.21	0.015	-2.41	0.012
GO:0098602	single organism cell adhesion	1.41	0.015	2.32	0.009
GO:0016337	single organismal cell-cell adhesion	1.53	0.015	2.29	0.009
GO:0007184	SMAD protein import into nucleus	-2.06	0.015	1.6	0.056
GO:0035725	sodium ion transmembrane transport	1.45	0.074	-2.04	0.01
GO:0001964	startle response	1.99	0.015	-2.26	0.01
GO:0048864	stem cell development	-2.03	0.015	1.67	0.009
GO:0042554	superoxide anion generation	1.45	0.216	2.05	0.009
GO:0006801	superoxide metabolic process	1.24	0.305	2.31	0.009
GO:0044403	symbiosis, encompassing mutualism through parasitism	1.43	0.015	2.34	0.009
GO:0007416	synapse assembly	2.23	0.015	-2.85	0.01
GO:0050808	synapse organization	2.09	0.015	-2.75	0.011
GO:0007268	synaptic transmission	2.5	0.015	-2.86	0.012
GO:0051932	synaptic transmission, GABAergic	1.88	0.015	-2.55	0.01
GO:0035249	synaptic transmission, glutamatergic	2.24	0.015	-2.9	0.01
GO:0016079	synaptic vesicle exocytosis	2.24	0.015	-2.73	0.01
GO:0097479	synaptic vesicle localization	2.08	0.015	-2.69	0.01
GO:0016082	synaptic vesicle priming	1.94	0.015	-2.21	0.01
GO:0036465	synaptic vesicle recycling	1.55	0.059	-2.12	0.01
GO:0048489	synaptic vesicle transport	2.04	0.015	-2.65	0.01
GO:0042088	T-helper 1 type immune response	1.74	0.024	2.4	0.009
GO:0042110	T cell activation	1.45	0.015	2.48	0.009
GO:0002286	T cell activation involved in immune response	2.09	0.015	2.23	0.009
GO:0070489	T cell aggregation	1.45	0.015	2.48	0.009
GO:0070231	T cell apoptotic process	-1.14	0.44	2.13	0.009
GO:0010818	T cell chemotaxis	2.04	0.015	1.92	0.009

GO:0030217	T cell differentiation	1.37	0.046	2.22	0.009
GO:0033077	T cell differentiation in thymus	0.99	0.642	2.05	0.009
GO:0002456	T cell mediated immunity	1.26	0.25	2.01	0.009
GO:0072678	T cell migration	2.15	0.015	2.3	0.009
GO:0042098	T cell proliferation	1.49	0.024	2.54	0.009
GO:0045058	T cell selection	1.15	0.459	2.2	0.009
GO:0042330	taxis	2.09	0.015	1.94	0.009
GO:0046653	tetrahydrofolate metabolic process	0.97	0.67	2.07	0.009
GO:0071594	thymocyte aggregation	0.99	0.642	2.05	0.009
GO:0048771	tissue remodeling	-1.42	0.049	2.35	0.009
GO:0002224	toll-like receptor signaling pathway	1.28	0.274	2.25	0.009
GO:0006412	translation	0.88	0.986	2.04	0.009
GO:0019226	transmission of nerve impulse	2.36	0.015	-2.27	0.01
GO:0042533	tumor necrosis factor biosynthetic process	1.9	0.024	2.27	0.009
GO:0032640	tumor necrosis factor production	1.71	0.024	2.67	0.009
GO:0071706	tumor necrosis factor superfamily cytokine production	1.74	0.015	2.67	0.009
GO:0042092	type 2 immune response	1.89	0.015	2.06	0.009
GO:0032606	type I interferon production	0.97	0.681	2.24	0.009
GO:0006636	unsaturated fatty acid biosynthetic process	-0.81	0.913	2.03	0.009
GO:0033559	unsaturated fatty acid metabolic process	-1.04	0.58	2.04	0.009
GO:0010573	vascular endothelial growth factor production	2.29	0.015	2.23	0.009
GO:0001944	vasculature development	-1.68	0.015	2.31	0.009
GO:0051648	vesicle localization	1.95	0.015	-2.41	0.01
GO:0046718	viral entry into host cell	-1.15	0.416	2.14	0.009
GO:0019058	viral life cycle	1.1	0.436	2.2	0.009
GO:0016032	viral process	-1.16	0.316	2.25	0.009
GO:0007632	visual behavior	1.41	0.148	-2.03	0.01
GO:0008542	visual learning	1.41	0.152	-2.05	0.01
GO:0090659	walking behavior	1.81	0.024	-2.06	0.01
GO:0060071	Wnt signaling pathway, planar cell polarity pathway	-2.21	0.015	1.67	0.023
GO:0042060	wound healing	-1.32	0.042	2.31	0.009

Supplementary Table 5: Overlap of GO terms (molecular function) with significant positive or negative enrichment in GSEA at either 6 or 24 hrs post-FUS (versus contralateral hemisphere) with ORA

Post-FUS Time Point	GO ID	GO Description	Gene Ratio	Background Ratio	Adjusted p-value
Upregulated genes 6 hrs Post-FUS	GO:0048020	CCR chemokine receptor binding	4/57	30/19509	0.0001
	GO:0008009	chemokine activity	6/57	36/19509	0.00000
	GO:0042379	chemokine receptor binding	6/57	48/19509	0.00000
	GO:0005125	cytokine activity	9/57	185/19509	0.00000
	GO:0005126	cytokine receptor binding	9/57	253/19509	0.00000
	GO:0001664	G-protein coupled receptor binding	8/57	255/19509	0.00004
	GO:0005102	receptor binding	16/57	1428/19509	0.0001
Downregulated genes 6 hrs Post-FUS	GO:0008509	anion transmembrane transporter activity	9/88	260/19509	0.0001
	GO:0005518	collagen binding	6/88	65/19509	0.0001
	GO:0005201	extracellular matrix structural constituent	4/88	38/19509	0.001
	GO:0005539	glycosaminoglycan binding	8/88	173/19509	0.0001
	GO:0008201	heparin binding	7/88	133/19509	0.0001
	GO:0008028	monocarboxylic acid transmembrane transporter activity	4/88	38/19509	0.001
	GO:0008514	organic anion transmembrane transporter activity	7/88	158/19509	0.0003
	GO:1901681	sulfur compound binding	11/88	224/19509	0.00000
	GO:0022857	transmembrane transporter activity	17/88	960/19509	0.0001
Upregulated genes 24 hrs Post-FUS	GO:0005215	transporter activity	17/88	1209/19509	0.001
	GO:0019956	chemokine binding	4/84	16/19509	0.0001
	GO:0019955	cytokine binding	6/84	87/19509	0.0002
	GO:00051021	receptor binding	19/84	1428/19509	0.0004

Supplementary Table 6: Overlap of GO terms (biological process) with significant positive or negative enrichment in GSEA at either 6 or 24 hrs post-FUS (versus contralateral hemisphere) with ORA

Post-FUS Time Point	GO ID	GO Description	Gene Ratio	Background Ratio	Adjusted P-value
Upregulated genes 6 hrs Post-FUS	GO:0002526	acute inflammatory response	5/59	125/19509	0.0004
	GO:0002460	adaptive immune response based on somatic recombination of immune receptors built from immunoglobulin superfamily domains	6/59	211/19509	0.0004
	GO:0001525	angiogenesis	8/59	391/19509	0.0003
	GO:0043277	apoptotic cell clearance	3/59	30/19509	0.001
	GO:0007610	behavior	16/59	788/19509	0.00000
	GO:0001568	blood vessel development	10/59	552/19509	0.0001
	GO:0048514	blood vessel morphogenesis	9/59	477/19509	0.0002
	GO:0072358	cardiovascular system development	11/59	937/19509	0.001
	GO:0001775	cell activation	11/59	799/19509	0.0003
	GO:0060326	cell chemotaxis	10/59	216/19509	0.00000
	GO:0016477	cell migration	15/59	1108/19509	0.00002
	GO:0008283	cell proliferation	19/59	1828/19509	0.00002
	GO:0045123	cellular extravasation	4/59	44/19509	0.0001
	GO:0071216	cellular response to biotic stimulus	11/59	236/19509	0
	GO:0071345	cellular response to cytokine stimulus	14/59	509/19509	0.00000
	GO:0071346	cellular response to interferon-gamma	5/59	70/19509	0.00004
	GO:0071347	cellular response to interleukin-1	8/59	96/19509	0.00000
	GO:0071396	cellular response to lipid	12/59	527/19509	0.00000
	GO:0071222	cellular response to lipopolysaccharide	11/59	206/19509	0
	GO:0071219	cellular response to molecule of bacterial origin	11/59	216/19509	0
	GO:0034614	cellular response to reactive oxygen species	6/59	144/19509	0.0001
	GO:0071356	cellular response to tumor necrosis factor	7/59	145/19509	0.00001
	GO:0070098	chemokine-mediated signaling pathway	6/59	67/19509	0.00000
	GO:0050755	chemokine metabolic process	3/59	13/19509	0.0001
	GO:0006935	chemotaxis	12/59	446/19509	0.00000
	GO:0002544	chronic inflammatory response	3/59	29/19509	0.001
	GO:0072359	circulatory system development	11/59	937/19509	0.001
	GO:0050890	cognition	7/59	282/19509	0.0003
	GO:0019221	cytokine-mediated signaling pathway	10/59	292/19509	0.00000
	GO:0001816	cytokine production	13/59	557/19509	0.00000
	GO:0050663	cytokine secretion	7/59	153/19509	0.00001
	GO:0006952	defense response	22/59	1191/19509	0
	GO:0048245	eosinophil chemotaxis	4/59	18/19509	0.00001
	GO:0072677	eosinophil migration	4/59	22/19509	0.00001
	GO:0071621	granulocyte chemotaxis	7/59	87/19509	0.00000
	GO:0097530	granulocyte migration	7/59	98/19509	0.00000
	GO:0048534	hematopoietic or lymphoid organ development	12/59	816/19509	0.0001
	GO:0030097	hemopoiesis	11/59	765/19509	0.0002
	GO:0002252	immune effector process	10/59	624/19509	0.0002
	GO:0006955	immune response	20/59	1030/19509	0
	GO:0002520	immune system development	14/59	862/19509	0.00001
	GO:0002376	immune system process	29/59	2040/19509	0
	GO:0006954	inflammatory response	16/59	540/19509	0
	GO:0045087	innate immune response	14/59	471/19509	0
	GO:0051702	interaction with symbiont	4/59	54/19509	0.0003
	GO:0044419	interspecies interaction between organisms	7/59	283/19509	0.0003
	GO:0007612	learning	6/59	148/19509	0.0001
	GO:0007611	learning or memory	7/59	251/19509	0.0001
	GO:0045321	leukocyte activation	10/59	678/19509	0.0004
	GO:0030595	leukocyte chemotaxis	9/59	159/19509	0.00000
	GO:0002521	leukocyte differentiation	9/59	456/19509	0.0001
	GO:0050900	leukocyte migration	11/59	253/19509	0.00000
	GO:0031663	lipopolysaccharide-mediated signaling pathway	5/59	47/19509	0.00001
	GO:0048247	lymphocyte chemotaxis	5/59	40/19509	0.00000
	GO:0072676	lymphocyte migration	5/59	65/19509	0.00003
	GO:0048246	macrophage chemotaxis	3/59	27/19509	0.001
	GO:0051851	modification by host of symbiont morphology or physiology	4/59	51/19509	0.0002

GO:0035821	modification of morphology or physiology of other organism	5/59	82/19509	0.0001
GO:0051817	modification of morphology or physiology of other organism involved in symbiotic interaction	5/59	76/19509	0.0001
GO:0002548	monocyte chemotaxis	5/59	45/19509	0.00001
GO:0002573	myeloid leukocyte differentiation	6/59	188/19509	0.0003
GO:0002444	myeloid leukocyte mediated immunity	5/59	82/19509	0.0001
GO:0097529	myeloid leukocyte migration	8/59	143/19509	0.00000
GO:0043901	negative regulation of multi-organism process	5/59	147/19509	0.001
GO:0030593	neutrophil chemotaxis	7/59	75/19509	0.00000
GO:0002446	neutrophil mediated immunity	3/59	28/19509	0.001
GO:1990266	neutrophil migration	7/59	84/19509	0.00000
GO:0006809	nitric oxide biosynthetic process	4/59	66/19509	0.0005
GO:0046209	nitric oxide metabolic process	4/59	70/19509	0.001
GO:0006909	phagocytosis	5/59	138/19509	0.001
GO:0002675	positive regulation of acute inflammatory response	3/59	30/19509	0.001
GO:0045766	positive regulation of angiogenesis	5/59	124/19509	0.0004
GO:0048520	positive regulation of behavior	8/59	148/19509	0.00000
GO:0030335	positive regulation of cell migration	10/59	383/19509	0.00001
GO:2000147	positive regulation of cell motility	10/59	394/19509	0.00001
GO:0008284	positive regulation of cell proliferation	11/59	854/19509	0.0005
GO:0051272	positive regulation of cellular component movement	10/59	403/19509	0.00001
GO:0050921	positive regulation of chemotaxis	8/59	120/19509	0.00000
GO:0001819	positive regulation of cytokine production	9/59	334/19509	0.00002
GO:0031349	positive regulation of defense response	10/59	252/19509	0.00000
GO:0045807	positive regulation of endocytosis	7/59	124/19509	0.00000
GO:0070374	positive regulation of ERK1 and ERK2 cascade	7/59	156/19509	0.00001
GO:1903708	positive regulation of hemopoiesis	8/59	165/19509	0.00000
GO:0002684	positive regulation of immune system process	19/59	690/19509	0
GO:0050729	positive regulation of inflammatory response	6/59	93/19509	0.00001
GO:0002690	positive regulation of leukocyte chemotaxis	8/59	78/19509	0
GO:1902107	positive regulation of leukocyte differentiation	7/59	131/19509	0.00000
GO:0002687	positive regulation of leukocyte migration	10/59	115/19509	0
GO:0040017	positive regulation of locomotion	10/59	414/19509	0.00001
GO:0002763	positive regulation of myeloid leukocyte differentiation	4/59	56/19509	0.0003
GO:0045429	positive regulation of nitric oxide biosynthetic process	4/59	41/19509	0.0001
GO:1904407	positive regulation of nitric oxide metabolic process	4/59	41/19509	0.0001
GO:0050766	positive regulation of phagocytosis	4/59	57/19509	0.0003
GO:1903428	positive regulation of reactive oxygen species biosynthetic process	4/59	48/19509	0.0002
GO:2000379	positive regulation of reactive oxygen species metabolic process	5/59	88/19509	0.0001
GO:0032103	positive regulation of response to external stimulus	10/59	263/19509	0.00000
GO:0048584	positive regulation of response to stimulus	16/59	1688/19509	0.0003
GO:1903036	positive regulation of response to wounding	6/59	141/19509	0.0001
GO:1904018	positive regulation of vasculature development	5/59	133/19509	0.001
GO:2001057	reactive nitrogen species metabolic process	4/59	74/19509	0.001
GO:0072593	reactive oxygen species metabolic process	6/59	232/19509	0.001
GO:0045765	regulation of angiogenesis	7/59	205/19509	0.00005
GO:0030334	regulation of cell migration	11/59	638/19509	0.0001
GO:2000145	regulation of cell motility	11/59	671/19509	0.0001
GO:0042127	regulation of cell proliferation	19/59	1501/19509	0.00000
GO:0051270	regulation of cellular component movement	11/59	749/19509	0.0002
GO:0050920	regulation of chemotaxis	8/59	161/19509	0.00000
GO:0001817	regulation of cytokine production	12/59	502/19509	0.00000
GO:0050707	regulation of cytokine secretion	6/59	134/19509	0.0001
GO:0031347	regulation of defense response	11/59	537/19509	0.00001
GO:1903706	regulation of hemopoiesis	8/59	322/19509	0.0001
GO:0002682	regulation of immune system process	20/59	1089/19509	0
GO:0050727	regulation of inflammatory response	7/59	248/19509	0.0001
GO:0043269	regulation of ion transport	10/59	621/19509	0.0002
GO:0002688	regulation of leukocyte chemotaxis	8/59	94/19509	0.00000
GO:1902105	regulation of leukocyte differentiation	7/59	231/19509	0.0001
GO:0002685	regulation of leukocyte migration	10/59	151/19509	0

	GO:0040012	regulation of locomotion	13/59	744/19509	0.00001
	GO:1901623	regulation of lymphocyte chemotaxis	3/59	19/19509	0.0003
	GO:0045428	regulation of nitric oxide biosynthetic process	4/59	56/19509	0.0003
	GO:0050764	regulation of phagocytosis	4/59	76/19509	0.001
	GO:1903426	regulation of reactive oxygen species biosynthetic process	4/59	68/19509	0.001
	GO:2000377	regulation of reactive oxygen species metabolic process	6/59	154/19509	0.0001
	GO:0032101	regulation of response to external stimulus	13/59	790/19509	0.00001
	GO:0010574	regulation of vascular endothelial growth factor production	4/59	30/19509	0.00004
	GO:1901342	regulation of vasculature development	7/59	222/19509	0.0001
	GO:0033198	response to ATP	3/59	31/19509	0.001
	GO:0009617	response to bacterium	20/59	608/19509	0
	GO:0009607	response to biotic stimulus	24/59	938/19509	0
	GO:0031960	response to corticosteroid	9/59	274/19509	0.00000
	GO:0034097	response to cytokine	22/59	644/19509	0
	GO:0043207	response to external biotic stimulus	24/59	909/19509	0
	GO:0010332	response to gamma radiation	5/59	64/19509	0.00003
	GO:0051384	response to glucocorticoid	9/59	254/19509	0.00000
	GO:0009629	response to gravity	3/59	18/19509	0.0003
	GO:0042542	response to hydrogen peroxide	6/59	154/19509	0.0001
	GO:0034341	response to interferon-gamma	5/59	93/19509	0.0001
	GO:0070555	response to interleukin-1	9/59	127/19509	0
	GO:0033993	response to lipid	22/59	1108/19509	0
	GO:0032496	response to lipopolysaccharide	19/59	403/19509	0
	GO:0055094	response to lipoprotein particle	3/59	17/19509	0.0002
	GO:0002237	response to molecule of bacterial origin	19/59	418/19509	0
	GO:0051707	response to other organism	24/59	907/19509	0
	GO:0000302	response to reactive oxygen species	8/59	237/19509	0.00001
	GO:0006950	response to stress	29/59	3260/19509	0.00000
	GO:0034612	response to tumor necrosis factor	8/59	169/19509	0.00000
	GO:0009611	response to wounding	11/59	728/19509	0.0001
	GO:0044708	single-organism behavior	8/59	477/19509	0.001
	GO:0044403	symbiosis, encompassing mutualism through parasitism	7/59	283/19509	0.0003
	GO:0042330	taxis	12/59	447/19509	0.00000
	GO:0010573	vascular endothelial growth factor production	4/59	32/19509	0.00005
	GO:0001944	vasculature development	10/59	576/19509	0.0001
Downregulated genes 6 hrs Post-FUS	GO:00713961	cellular response to lipid	12/94	527/19509	0.001
	GO:0030198	extracellular matrix organization	9/94	197/19509	0.0001
	GO:0043062	extracellular structure organization	9/94	198/19509	0.0001
	GO:00339931	response to lipid	23/94	1108/19509	0.00000
Upregulated genes 24 hrs Post-FUS	GO:0002253	activation of immune response	14/90	267/19509	0
	GO:0002250	adaptive immune response	12/90	280/19509	0.00000
	GO:00024601	adaptive immune response based on somatic recombination of immune receptors built from immunoglobulin superfamily domains	8/90	211/19509	0.0001
	GO:0019882	antigen processing and presentation	7/90	114/19509	0.00002
	GO:0019884	antigen processing and presentation of exogenous antigen	4/90	27/19509	0.0001
	GO:0002478	antigen processing and presentation of exogenous peptide antigen	4/90	22/19509	0.0001
	GO:0019886	antigen processing and presentation of exogenous peptide antigen via MHC class II	3/90	14/19509	0.0005
	GO:0048002	antigen processing and presentation of peptide antigen	6/90	69/19509	0.00002
	GO:0002495	antigen processing and presentation of peptide antigen via MHC class II	5/90	20/19509	0.00000
	GO:0002504	antigen processing and presentation of peptide or polysaccharide antigen via MHC class II	6/90	25/19509	0.00000
	GO:0019724	B cell mediated immunity	6/90	103/19509	0.0001
	GO:0050853	B cell receptor signaling pathway	4/90	40/19509	0.0005
	GO:00017751	cell activation	28/90	799/19509	0

GO:0002263	cell activation involved in immune response	10/90	194/19509	0.00000
GO:00603261	cell chemotaxis	11/90	216/19509	0.00000
GO:00164771	cell migration	18/90	1108/19509	0.00005
GO:00082831	cell proliferation	26/90	1828/19509	0.00000
GO:00712161	cellular response to biotic stimulus	11/90	236/19509	0.00000
GO:00713962	cellular response to lipid	13/90	527/19509	0.00002
GO:00712221	cellular response to lipopolysaccharide	9/90	206/19509	0.00001
GO:00712191	cellular response to molecule of bacterial origin	9/90	216/19509	0.00002
GO:00069351	chemotaxis	13/90	446/19509	0.00000
GO:0006956	complement activation	4/90	44/19509	0.001
GO:00018161	cytokine production	13/90	557/19509	0.00003
GO:00069521	defense response	34/90	1191/19509	0
GO:0042742	defense response to bacterium	7/90	221/19509	0.001
GO:0098542	defense response to other organism	10/90	467/19509	0.001
GO:00716211	granulocyte chemotaxis	7/90	87/19509	0.00001
GO:00975301	granulocyte migration	7/90	98/19509	0.00001
GO:0034109	homotypic cell-cell adhesion	16/90	464/19509	0.00000
GO:0006959	humoral immune response	8/90	139/19509	0.00001
GO:0002455	humoral immune response mediated by circulating immunoglobulin	4/90	40/19509	0.0005
GO:00022521	immune effector process	21/90	624/19509	0
GO:00069551	immune response	37/90	1030/19509	0
GO:0002429	immune response-activating cell surface receptor signaling pathway	7/90	119/19509	0.00003
GO:0002757	immune response-activating signal transduction	9/90	218/19509	0.00002
GO:0002768	immune response-regulating cell surface receptor signaling pathway	8/90	137/19509	0.00001
GO:0002764	immune response-regulating signaling pathway	10/90	238/19509	0.00001
GO:00023761	immune system process	44/90	2040/19509	0
GO:0016064	immunoglobulin mediated immune response	6/90	101/19509	0.0001
GO:00069541	inflammatory response	17/90	540/19509	0.00000
GO:0002437	inflammatory response to antigenic stimulus	5/90	63/19509	0.0002
GO:00450871	innate immune response	19/90	471/19509	0
GO:00453211	leukocyte activation	24/90	678/19509	0
GO:0002366	leukocyte activation involved in immune response	10/90	192/19509	0.00000
GO:0070486	leukocyte aggregation	14/90	397/19509	0.00000
GO:0007159	leukocyte cell-cell adhesion	14/90	426/19509	0.00000
GO:00305951	leukocyte chemotaxis	9/90	159/19509	0.00000
GO:0043299	leukocyte degranulation	5/90	64/19509	0.0002
GO:0002443	leukocyte mediated immunity	11/90	280/19509	0.00000
GO:00509001	leukocyte migration	11/90	253/19509	0.00000
GO:0070661	leukocyte proliferation	13/90	266/19509	0.00000
GO:0046649	lymphocyte activation	18/90	564/19509	0
GO:0071593	lymphocyte aggregation	13/90	390/19509	0.00000
GO:0046651	lymphocyte proliferation	13/90	254/19509	0.00000
GO:0032943	mononuclear cell proliferation	13/90	256/19509	0.00000
GO:0002275	myeloid cell activation involved in immune response	7/90	69/19509	0.00000
GO:0002274	myeloid leukocyte activation	9/90	164/19509	0.00000
GO:00024441	myeloid leukocyte mediated immunity	7/90	82/19509	0.00000
GO:00975291	myeloid leukocyte migration	8/90	143/19509	0.00001
GO:0050866	negative regulation of cell activation	8/90	158/19509	0.00002
GO:0002683	negative regulation of immune system process	10/90	373/19509	0.0001
GO:0002695	negative regulation of leukocyte activation	8/90	142/19509	0.00001
GO:1903038	negative regulation of leukocyte cell-cell adhesion	5/90	96/19509	0.001
GO:0051250	negative regulation of lymphocyte activation	6/90	124/19509	0.0003
GO:0050868	negative regulation of T cell activation	5/90	91/19509	0.001
GO:00305931	neutrophil chemotaxis	7/90	75/19509	0.00000
GO:19902661	neutrophil migration	7/90	84/19509	0.00000
GO:00026751	positive regulation of acute inflammatory response	4/90	30/19509	0.0002
GO:0002821	positive regulation of adaptive immune response	5/90	82/19509	0.001
GO:00457661	positive regulation of angiogenesis	6/90	124/19509	0.0003
GO:00485201	positive regulation of behavior	6/90	148/19509	0.001
GO:0022409	positive regulation of cell-cell adhesion	8/90	186/19509	0.00005
GO:0045785	positive regulation of cell adhesion	10/90	334/19509	0.0001
GO:00303351	positive regulation of cell migration	12/90	383/19509	0.00001

GO:20001471	positive regulation of cell motility	12/90	394/19509	0.00001
GO:00512721	positive regulation of cellular component movement	12/90	403/19509	0.00001
GO:00509211	positive regulation of chemotaxis	6/90	120/19509	0.0003
GO:00018191	positive regulation of cytokine production	11/90	334/19509	0.00001
GO:00313491	positive regulation of defense response	9/90	252/19509	0.00005
GO:0034112	positive regulation of homotypic cell-cell adhesion	7/90	151/19509	0.0001
GO:0050778	positive regulation of immune response	18/90	412/19509	0
GO:00026841	positive regulation of immune system process	23/90	690/19509	0
GO:00507291	positive regulation of inflammatory response	6/90	93/19509	0.0001
GO:1903039	positive regulation of leukocyte cell-cell adhesion	7/90	152/19509	0.0001
GO:00026871	positive regulation of leukocyte migration	6/90	115/19509	0.0002
GO:00400171	positive regulation of locomotion	12/90	414/19509	0.00001
GO:00321031	positive regulation of response to external stimulus	11/90	263/19509	0.00000
GO:00485841	positive regulation of response to stimulus	28/90	1688/19509	0.00000
GO:19030361	positive regulation of response to wounding	7/90	141/19509	0.0001
GO:0050870	positive regulation of T cell activation	6/90	146/19509	0.001
GO:0032760	positive regulation of tumor necrosis factor production	6/90	61/19509	0.00001
GO:1903557	positive regulation of tumor necrosis factor superfamily cytokine production	6/90	62/19509	0.00001
GO:19040181	positive regulation of vasculature development	6/90	133/19509	0.0005
GO:0002673	regulation of acute inflammatory response	5/90	58/19509	0.0001
GO:0002819	regulation of adaptive immune response	6/90	126/19509	0.0004
GO:00457651	regulation of angiogenesis	7/90	205/19509	0.001
GO:0002577	regulation of antigen processing and presentation	3/90	17/19509	0.001
GO:0022407	regulation of cell-cell adhesion	11/90	333/19509	0.00001
GO:0050865	regulation of cell activation	13/90	430/19509	0.00000
GO:0030155	regulation of cell adhesion	13/90	581/19509	0.00005
GO:00303341	regulation of cell migration	13/90	638/19509	0.0001
GO:20001451	regulation of cell motility	13/90	671/19509	0.0002
GO:00421271	regulation of cell proliferation	19/90	1501/19509	0.001
GO:00512701	regulation of cellular component movement	13/90	749/19509	0.001
GO:00018171	regulation of cytokine production	13/90	502/19509	0.00001
GO:00313471	regulation of defense response	12/90	537/19509	0.0001
GO:0034110	regulation of homotypic cell-cell adhesion	10/90	257/19509	0.00001
GO:0002920	regulation of humoral immune response	4/90	37/19509	0.0004
GO:0002697	regulation of immune effector process	10/90	378/19509	0.0002
GO:0050776	regulation of immune response	21/90	525/19509	0
GO:00026821	regulation of immune system process	26/90	1089/19509	0
GO:00507271	regulation of inflammatory response	8/90	248/19509	0.0003
GO:0002694	regulation of leukocyte activation	12/90	396/19509	0.00001
GO:1903037	regulation of leukocyte cell-cell adhesion	10/90	245/19509	0.00001
GO:00026851	regulation of leukocyte migration	6/90	151/19509	0.001
GO:0070663	regulation of leukocyte proliferation	10/90	197/19509	0.00000
GO:00400121	regulation of locomotion	14/90	744/19509	0.0001
GO:0051249	regulation of lymphocyte activation	11/90	335/19509	0.00001
GO:0050670	regulation of lymphocyte proliferation	10/90	189/19509	0.00000
GO:0032944	regulation of mononuclear cell proliferation	10/90	191/19509	0.00000
GO:0002886	regulation of myeloid leukocyte mediated immunity	4/90	44/19509	0.001
GO:00321011	regulation of response to external stimulus	15/90	790/19509	0.0001
GO:1903034	regulation of response to wounding	9/90	369/19509	0.001
GO:0050863	regulation of T cell activation	9/90	235/19509	0.00003
GO:0042129	regulation of T cell proliferation	8/90	139/19509	0.00001
GO:0032680	regulation of tumor necrosis factor production	7/90	107/19509	0.00002
GO:1903555	regulation of tumor necrosis factor superfamily cytokine production	7/90	109/19509	0.00002
GO:19013421	regulation of vasculature development	7/90	222/19509	0.001
GO:00096171	response to bacterium	14/90	608/19509	0.00002
GO:00096071	response to biotic stimulus	21/90	938/19509	0.00000
GO:00340971	response to cytokine	14/90	644/19509	0.00003
GO:00432071	response to external biotic stimulus	21/90	909/19509	0.00000
GO:00343411	response to interferon-gamma	5/90	93/19509	0.001
GO:00339932	response to lipid	19/90	1108/19509	0.00001
GO:00324961	response to lipopolysaccharide	10/90	403/19509	0.0002
GO:00022371	response to molecule of bacterial origin	11/90	418/19509	0.0001
GO:00517071	response to other organism	21/90	907/19509	0.00000

GO:00069501	response to stress	43/90	3260/19509	0
GO:00096111	response to wounding	16/90	728/19509	0.00001
GO:0098602	single organism cell adhesion	20/90	708/19509	0
GO:0016337	single organismal cell-cell adhesion	19/90	655/19509	0
GO:0042110	T cell activation	13/90	389/19509	0.00000
GO:0070489	T cell aggregation	13/90	389/19509	0.00000
GO:0042098	T cell proliferation	11/90	174/19509	0.00000
GO:00423301	taxis	13/90	447/19509	0.00000
GO:0032640	tumor necrosis factor production	7/90	108/19509	0.00002
GO:0071706	tumor necrosis factor superfamily cytokine production	7/90	111/19509	0.00002

Supplementary Table 6: Differential expression of selected genes at 6 and 24 hrs post-FUS as measured by microarray analysis and qRT-PCR

	**Log2 Fold Change Compared to Contralateral Hemisphere (adjusted p-value)**			
	**6 hrs Post-FUS**		**24 hrs Post-FUS**	
**Gene Symbol**	**Microarray**	**qRT-PCR**	**Microarray**	**qRT-PCR**
Abcb1a	**-1.14 (0.044)**	**-3.14 (0.049)**	0.08 (0.928)	0.68 (0.394)
Ccl2	**4.73 (<0.001)**	**6.16 (0.021)**	1.70 (0.086)	2.79 (0.255)
Cd74	0.34 (0.741)	0.23 (0.835)	**2.64 (0.010)**	**3.99 (0.001)**
Gfap	0.29 (0.479)	-0.17 (0.762)	**1.54 (0.003)**	**2.05 (<0.001)**
Itgb2	0.47 (0.479)	0.36 (0.613)	**1.38 (0.035)**	**1.77 (0.011)**
Lcn2	**2.02 (0.008)**	**2.16 (0.036)**	**2.91 (0.003)**	**4.41 (<0.001)**
Serpine1	**2.37 (0.037)**	**3.42 (0.013)**	1.97 (0.092)	**3.07 (0.043)**
Slc22a6	**-1.83 (0.003)**	**-2.98 (0.001)**	0.18 (0.801)	0.51 (0.386)
